# Novel hydroxyl carboximates derived from *β*-elemene: design, synthesis and anti-tumour activities evaluation

**DOI:** 10.1080/14756366.2022.2117314

**Published:** 2022-09-06

**Authors:** Yuan Gao, Nian-Dong Mao, Hao Che, Li Xu, Renren Bai, Li-Wei Wang, Xiang-Yang Ye, Tian Xie

**Affiliations:** a School of Pharmacy, Hangzhou Normal University, Hangzhou, China; b Key Laboratory of Elemene Class Anti-Cancer Chinese Medicine of Zhejiang Province, Hangzhou, China; c Engineering Laboratory of Development and Application of Traditional Chinese Medicine from Zhejiang Province, Hangzhou, China; d Collaborative Innovation Center of Chinese Medicines from Zhejiang Province, Hangzhou, China; e Institute of Chinese Materia Medical, Shanghai University of Traditional Chinese Medicine, Shanghai, China

**Keywords:** *β*-elemene, hydroxyl carboximate derivatives, anti-tumour drugs, lung cancer

## Abstract

A series of novel *N*-alkyl-*N*-hydroxyl carboximates derived from *β*-elemene were fortuitously discovered. Most of them showed more potent anti-proliferative activities than their lead compound *β*-elemene (**1**). Notably, compound **11i** exhibited significant inhibitory effects on the proliferation of three lung cell lines (H1975, A549 and H460) and several other tumour cell lines (H1299, U87MG, MV4-11, and SU-DHL-2). Preliminary mechanistic studies revealed that compound **11i** could significantly induce cell apoptosis. Further *in vivo* study in the H460 xenograft mouse model validated the anti-tumour activities of **11i** with a greater tumour growth inhibition (TGI, 68.3%) than *β*-elemene and SAHA (50.1% and 55.9% respectively) at 60 mg/kg ip dosing, without obvious body weight loss and toxicity. Thus, such *N*-alkyl-*N*-hydroxyl carboximate class of compounds exemplified as **11i** demonstrated potent anticancer activities both *in vitro* and *in vivo*, and should warrant further investigation for potential anticancer therapy.

## Graphical Abstract

**Figure:**
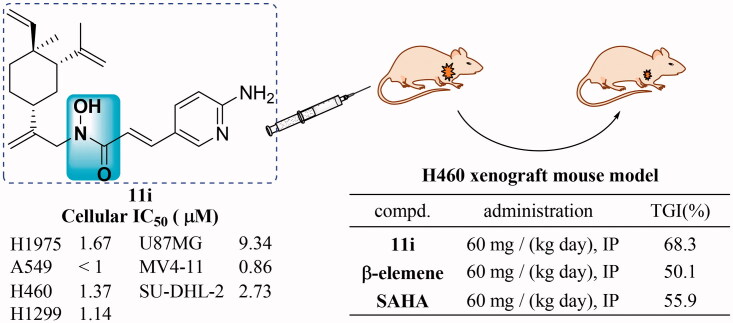

## 1. Introduction

**Figure 1.**
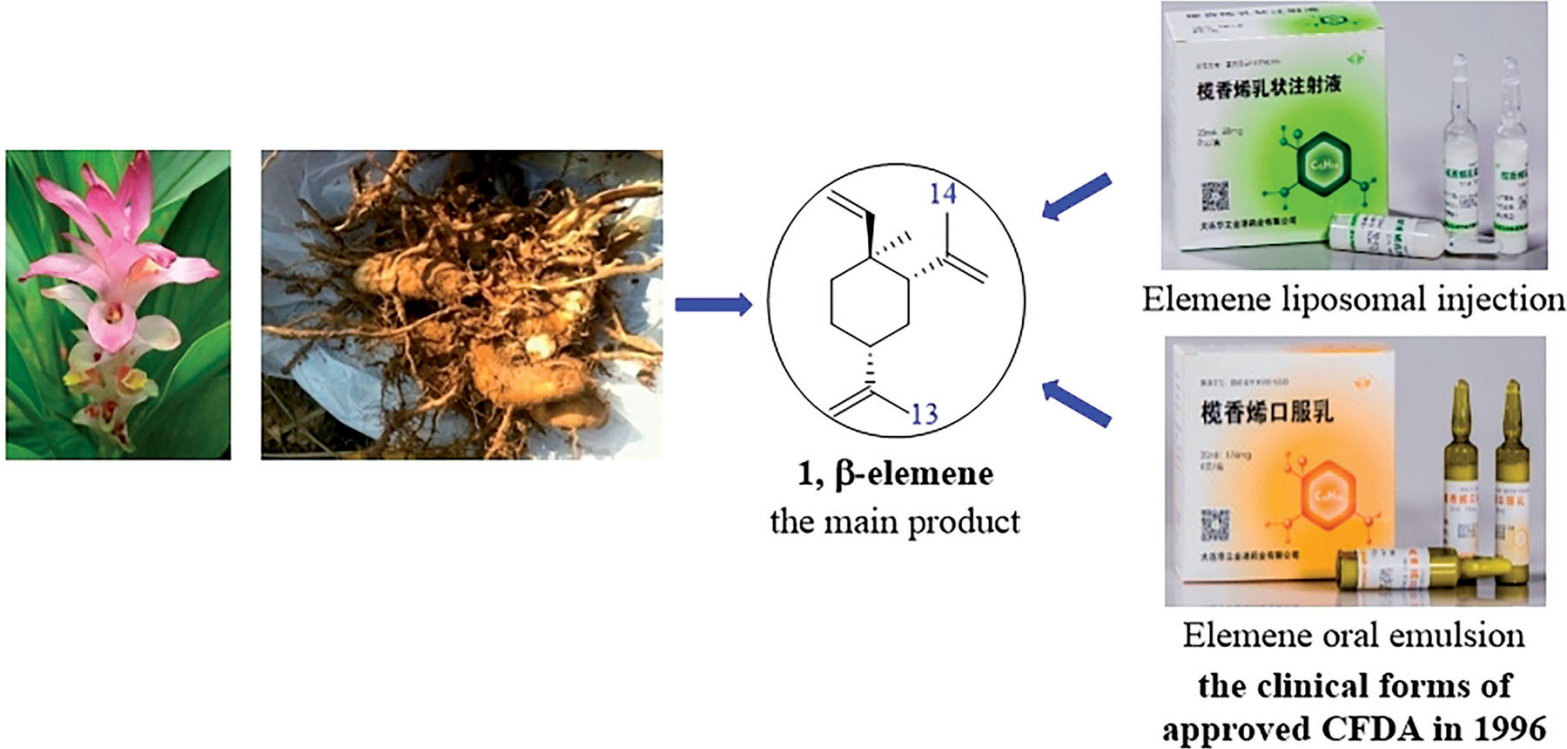
The source, structure and approved clinical forms of *β*-elemene.

Elemene extract obtained from the rhizome of *Curcuma wenyujin c*omposes of a mixture of sesquiterpene. Among these elemene isomers, *β*-elemene is the most abundant one and is responsible for elemene’s anti-tumour activities (Figure 1)^1^^,^^2^. In fact, elemene liposomal injection and elemene oral emulsion have been approved by the Chinese Food and Drug Administration (CFDA) for the treatment of various human cancers (Figure 1)^3^^,^^4^. Pharmacologically, *β*-elemene suppresses tumour cell growth *via* diverse effects including induction of apoptosis, autophagy and cell cycle arrest, and intervention of cell proliferation and migration^5–8^. Scientists also discovered that the combination of *β*-elemene with other immune drugs could enhance the body’s immune response to tumours^9^. Despite these attractive anticancer properties, the poor water solubility and moderate anti-tumour activities limit the maximisation of their clinical applications.

**Figure 2.**
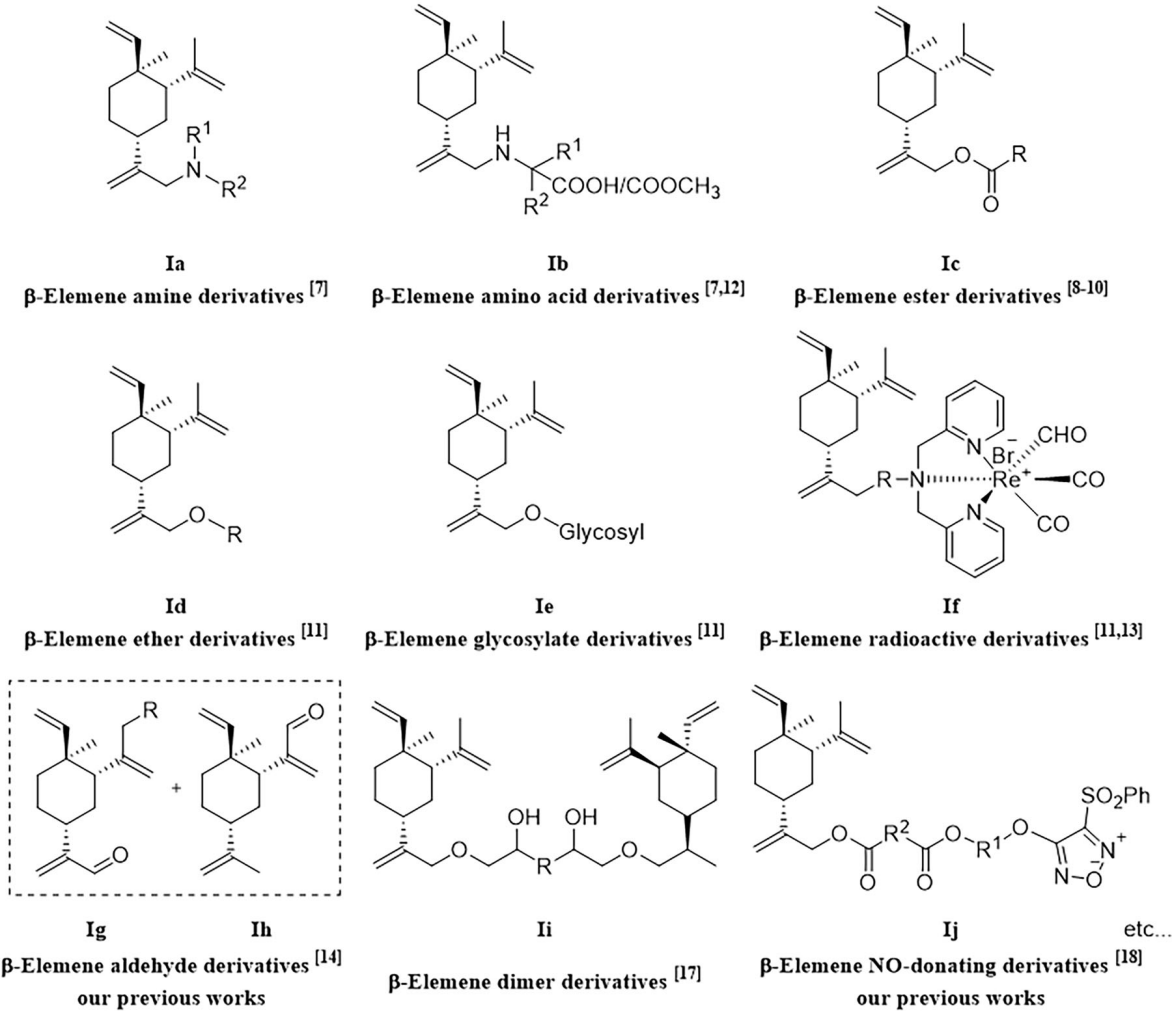
Classification of *β*-elemene derivatives reported in the literature.

To improve the water solubility and the anti-tumour efficacy of *β*-elemene, scientists from various research groups including us have worked on the structural modifications of *β*-elemene from then to now^10–17^. Based on the characteristics of functional groups, *β*-elemene derivatives could be divided into the following classes: amines, esters, amino acid derivatives, ethers, alcohols, glycosides and organometallic compounds, etc^18^, as shown in Figure 2. In recent years, the dimer derivatives^19^^,^^20^ (Figure 2, **Ii**) and NO donor derivatives^21^^,^^22^ (Figure 2, **Ij**) of *β*-elemene were investigated and showed promising biological activities. However, the hybrid drugs derived from *β*-elemene and histone deacetylase inhibitors (HDACi) were rarely studied and reported.

**Figure 3.**
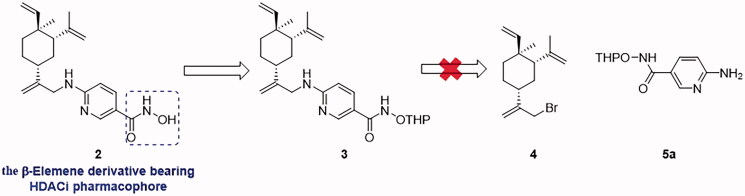
The designed hybrid compound **2** and the proposed retro-synthesis.

Our previous work in multi-targeting drug discovery^23^^,^^24^ prompted us to design the hybrid molecules of *β*-elemene by incorporating HDACi pharmacophore (hydroxamic acid). Thus, compound **2** was designed for such a purpose (Figure 3). Based on the retro-synthesis analysis, the key precursor **3** could be obtained from allylic bromide **4** and THP-protected hydroxyl carboximate **5a** (Figure 3). Unfortunately, the displacement reaction of **4** and **5a** produced two products; none of them was consistent with compound **3** despite of having the same molecular weight. Further investigation of the displacement products leads us to identify novel hydroxyl carboximate class compounds exhibiting potent anti-tumour activities. Herein, we wish to report this class of compounds, including their discovery, synthesis, and structure-activity relationship as well as *in vivo* anti-tumour efficacy.

## 2. Results and discussion

### 2.1. Discovery of novel N-alkyl-N-hydroxyl carboximates

**Figure 4.**
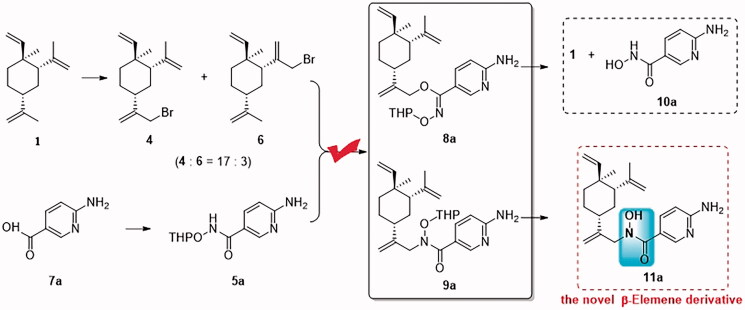
The discovery of *N*-alkyl-*N*-hydroxyl carboximate derivatives of *β*-elemene.

The discovery of *N*-alkyl-*N*-hydroxyl carboximate **11a** (Figure 4) was an accidental process. The hybrid compound, such as **2** was originally designed from *β*-elemene (**1**) and Suberoylanilide hydroxamic acid (SAHA or Vorinostat, a histone deacetylases (HDACs) inhibitor approved in 2006). The intention of reacting the amino group at the 2-position of pyridyl with allylic bromide **4** apparently did not go in the desired direction as we expected (Figure 3). Indeed, the alkylation produced two products with very close polarity on the TLC plate. They were separable only after multiple cycles of flash column chromatography in about 1:2 ratio (more polar product *vs* less polar compound). Structure elucidations of these two products were not straightforward even though several 2D NMR experiments were performed. This was due to many overlapping ^1^H NMR signals for both of them. Therefore, they were independently taken into the THP-deprotection step, and the products were characterised respectively. The less polar compound was deprotected to afford **1** and **10a** while the more polar compound to afford **11a**. The structure elucidations of **1**, **10a** and **11a** helped us to postulate the two products in the alkylation step. This became explainable that **9a** (R_f_ values 0.3 in TLC plate (petroleum ether-ethyl acetate 2:3, v/v)) was derived from the alkylation at the nitrogen atom of carboximate (more polar compound), while **8a** (R_f_ values 0.4 in TLC plate (petroleum ether-ethyl acetate 2:3, v/v)) was derived from the alkylation at the oxygen atom of the tautomer of carboximate (enol tautomerization form, less polar compound). The novel compound **11a** showed potent anticancer activities against three lung tumour lines (H1975, A549 and H460) *in vitro* anti-proliferative assay.

### 2.2. Chemistry

**Scheme 1.**
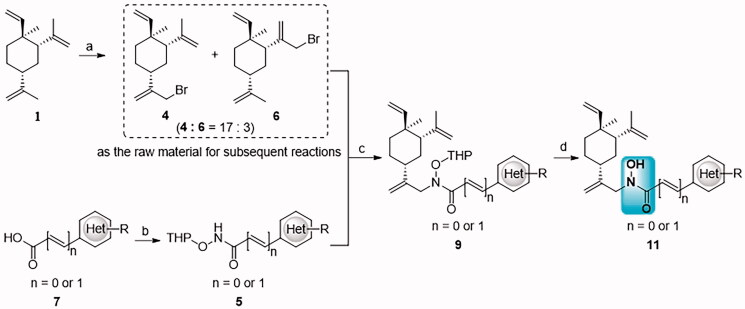
The proposed synthetic route for *N*-alkyl-*N*-hydroxyl carbomixate derivatives of *β*-elemene. Reaction conditions and reagents: (a) NBS, CH_3_CO_2_H, 0 °C to rt, 9 h; (b) EDCI, HOBT, DIPEA, DMF, NH_2_OTHP, rt, 5 h; (c) Cs_2_CO_3_, DMF, 60 °C, overnight; (d) TsOH·H_2_O, CH_3_OH, rt, 8 h.

The interesting biological activities and the easy accessibility of novel compound **11a** encouraged us to investigate more on *N*-alkyl-*N*-hydroxyl carboximate class analogs of *β*-elemene. In order to accomplish the work, we first needed to prepare 13-Br-*β*-elemene (**4**) in a workable amount (Scheme 1). Compound **4** was previously prepared from the corresponding allylic alcohol by Xu et al.^19^ using NBS/Ph_3_P condition. This route not only added one extra step of alcohol preparation but also required tedious separation of close related isomer in allylic alcohols. We worked out an alternative route of direct bromination at the 13-position (one of the allylic positions) of **1**. By following the procedure in our patent application^25^, compound **4** was prepared in 30.1% yield with good purity, enough for the next step displacement reaction. The minor isomer 14-Br-*β*-elemene (**6**) did not interfere with the following step reaction.

As mentioned above, the displacement of THP-protected *N*-hydroxyl carboximate with allylic bromide **4** yielded two isomers (i.e. **8** and **9**), close to each other on the TLC plate due to the similarity in polarity. Attempt to separate them using dichloromethane (DCM) and methanol (MeOH) mixed solvent in flash column chromatography did not give a satisfactory result. After several tries, we figured out the separation of these two isomers could be achieved by using petroleum ether (PE)-ethyl acetate (EA) as eluent in silica gel column for 2–3 separation cycles.

The removal of the THP group of **9** to afford compound **11** would be easily achieved using acid-catalyzed deprotection conditions. An attempt to purify **11** in silica gel column chromatography using a methanol-dichloromethane system proved to be unsuccessful. Finally, reversed-phase (C18) column chromatography was applied for the separation to afford pure compound **11** in good yields.

**Scheme 2.**
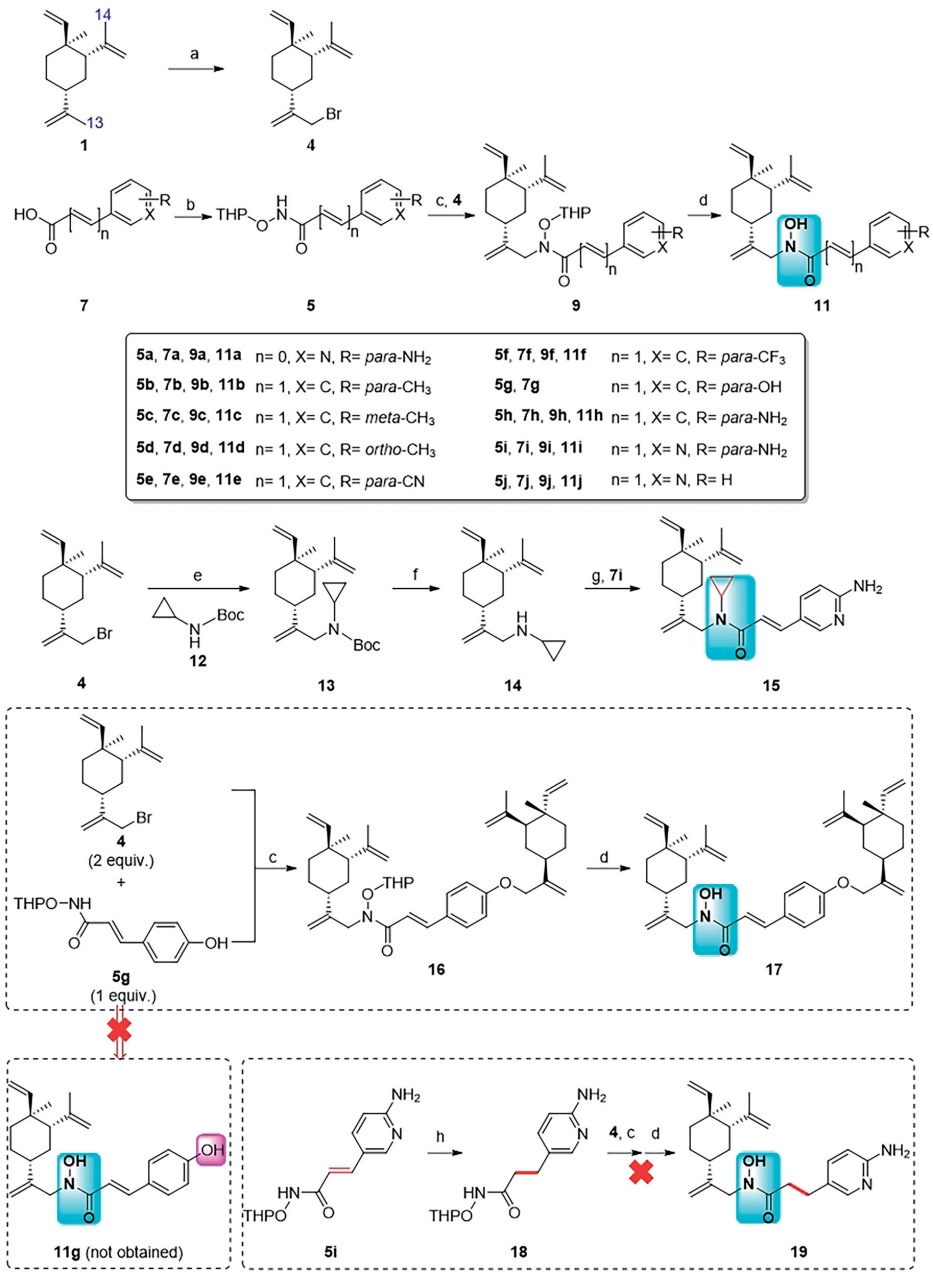
Synthetic routes of the target compounds **11a**–**11f**, **11h**–**11j**, **15** and **17**. Reaction conditions and reagents: (a) NBS, CH_3_CO_2_H, 0 °C to rt, 9 h, 30.1%; (b) EDCI, HOBT, DIPEA, DMF, NH_2_OTHP, rt, 5 h, 65.0–91.3%; (c) Cs_2_CO_3_, DMF, **4**, 60 °C, overnight, 15.4–31.0%; (d) TsOH·H_2_O, CH_3_OH, rt, 8 h, 50.6–94.0%; (e) Cs_2_CO_3_, DMF, **12**, 60 °C, overnight, 90.1%; (f) HCl-Dioxane (4 M), CH_3_OH, 8 h, > 90%; (g) EDCI, HOBT, DIPEA, DMF, **7i**, rt, 5 h, 50.3%; (h) 10% Pd/C, H_2_, CH_3_OH, rt, 3 h, 40.7%.

Thus the proposed synthetic route for *N*-alkyl-*N*-hydroxyl carbomixates **11** (Scheme 1) is now practical for medicinal chemistry purposes. The synthesis steps for the target compound **11** were detailed in Scheme 2. The bromination of *β*-elemene (**1**) was achieved using NBS in acetic acid in 30.1% yields with good selectivity of 13-position over 14-position (**4 **: **6 **=** **17 : 3). Small amount of isomer **6** did not interfere with the following step reaction. Subsequently, the intermediates **5a**–**5j** could be easily prepared from the corresponding carboxylic acids **7a**–**7j** and *O*-(tetrahydro-2*H*-pyran-2-yl)hydroxylamine (NH_2_OTHP) under the standard amide coupling reaction conditions such as HOBt/EDCI/DIPEA. The group “R” on different positions of the aromatic ring of carboxylic acids **7a**–**7j** were designed to examine the substituent effects based on the principle of bioisosterism in medicinal chemistry (eg: −NH_2_ *vs* −OH, −CH_3_; −CN *vs* −CF_3_) (Scheme 2). The displacement reaction between **5a**–**5f** and **5h**–**5j** and allylic bromide **4** could be achieved under the condition of Cs_2_CO_3_/DMF at 60 °C overnight. Finally, the THP-deprotection of **9** under acidic condition TsOH·H_2_O/MeOH at room temperature, afforded the target compounds **11a**–**11f** and **11h**–**11j** in 50.6% to 94.0% yields.

In addition, compound **15** was designed for comparison with compound **11i**. The synthetic route for **11** was modified in the allylic bromide displacement step using *N*-Boc-cyclopropylamine (**12**) instead of **5**, as shown in Scheme 1. The resulting intermediate **13** underwent a Boc-deprotection process, yielding compound **14**. Subsequent condensation of **14** with **7i** afforded the desired product **15**.

**Scheme 3.**
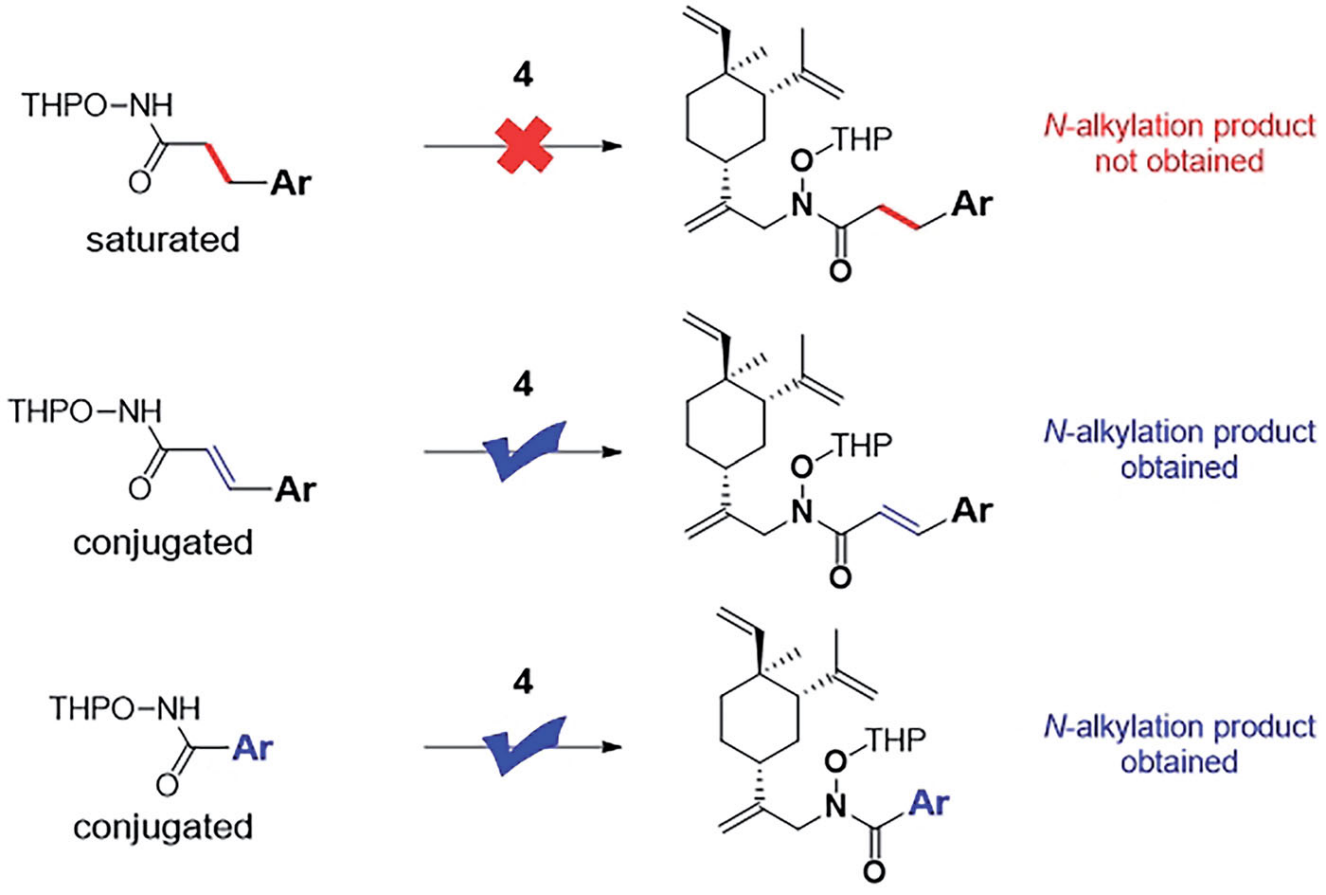
A Conjugated system to THPO-NH-C(O)- was required for *N*-alkylation.

Intermediate **5g** (R = *para*-OH) possesses at least three reactive centres which could potentially react with intermediate **4**. In fact, the designed analog **11g** was unable to obtain using the above synthetic route. During the displacement step, double alkylation occurred and compound **16** was isolated. This was explainable that Cs_2_CO_3_ was capable to deprotonate both phenolic proton and nitrogen proton of THPO-NH-C(O)- group. The resulting dianion attacked the Br-bearing allylic carbon of **4** to facilitate the double alkylation. The final deprotection of **16** gave the novel dimer derivative **17**, which was also subjected to biological testing. It should be noted that compound **19**, an analog of **11i** with saturated *α*,*β*- position of carboximate group, could not be synthesised from **18** and **4** using the above synthetic route. Apparently, the alkylation at the nitrogen of THPO-NH-C(O)- did not proceed as expected. By comparing with the majority of substrates **5** (no matter n is 0 or 1), one could postulate that the conjugated component (C−C double bond or aryl group) directly attaching to THPO-NH-C(O)- was necessitated for *N*-alkylation (Scheme 3) to generate novel *N*-alkyl-*N*-hydroxyl carboximate derivatives of *β*-elemene.

### 2.3. Biological evaluation

#### 2.3.1. *In vitro* anti-tumour activities

The *in vitro* anti-tumour activities of all the compounds against three human lung tumour cell lines H1975, A549, and H460 were determined (Table 1). To investigate the effects of substituted positions of the phenyl ring on anti-proliferative activities, compounds **11b**–**11d** were synthesised. The results suggested that the substitution position on the phenyl ring has little effect on the activities (i.e. methyl group at *ortho*-, *meta*-, and *para*- positions). We chose *para*-substituted on the phenyl ring for further analogs exploration. Compounds **11e**–**11f** and **11h** were synthesised and compared with **11b**. The results revealed that replacing the methyl group on the phenyl ring of **11b** with CN, CF_3_ and NH_2_ did not have much influence on tumour inhibition effects (**11e**, **11f** and **11h**). Considering the price of precursor drugs with CN and CF_3_, the **11h** displayed 1–3 fold stronger inhibitory activities against tumour cell lines compared to **11b**, so we used compound **11h** as a new starting point for further structural modifications. It should be noted that the *para*-OH analog **11g** was designed but we were unable to obtain the compound. The displacement step of **5g** with **4** yielded **16**, a double alkylation product at both N atom and phenolic oxygen, despite of the presence of the excess amount of **4** (2 equivalent). The deprotection of **16** gave compound **17**. Unfortunately, **17** showed poor inhibitory effects against three human lung tumour cell lines (IC_50_ > 30 μM).

**Table 1.** The structures and corresponding IC_50_ values of the target compounds against three lung tumour cell lines.

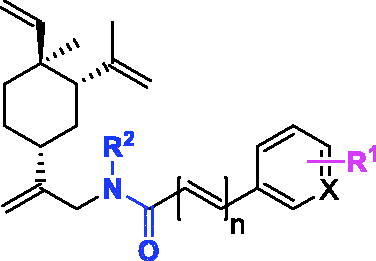								
Compd.	n	X	R^1^		R^2^	IC_50_ (µM)^a^		
						H1975	A549	H460
**11a**	0	N	*para*	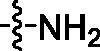	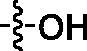	26.81 ± 0.11	16.06 ± 0.13	16.38 ± 1.50
**11b**	1	C	*para*	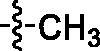	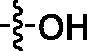	26.92 ± 0.71	8.70 ± 0.07	7.46 ± 1.12
**11c**	1	C	*meta*	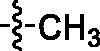	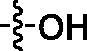	>30	7.64 ± 0.82	5.03 ± 0.34
**11d**	1	C	*ortho*	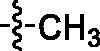	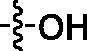	25.35 ± 2.77	6.12 ± 0.15	3.59 ± 0.16
**11e**	1	C	*para*	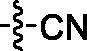	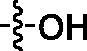	4.32 ± 0.40	3.06 ± 0.15	2.05 ± 0.18
**11f**	1	C	*para*	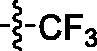	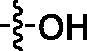	13.65 ± 0.23	7.38 ± 1.80	3.81 ± 0.09
**17**	1	C	*para*	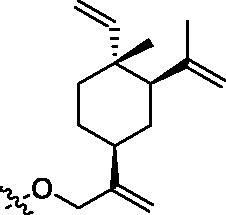	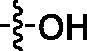	>30	>30	>30
**11h**	1	C	*para*	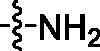	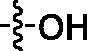	7.21 ± 2.50	6.35 ± 1.76	3.49 ± 0.90
**11i**	1	N	*para*	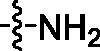	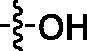	1.67 ± 0.11	<1	1.37 ± 0.04
**11j**	1	N	—	H	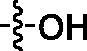	17.56 ± 3.14	2.51 ± 0.26	5.71 ± 0.37
**15**	1	N	*para*	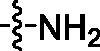	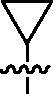	>30	9.28 ± 2.50	>30
**1**	—	—	—	—	—	>100	>100	>100
**SAHA**	—	—	—	—	—	1.34 ± 0.29	1.82 ± 0.04	1.63 ± 0.05

^a^Data represent the mean value from at least three independent experiments. SAHA was used as positive control for *in vitro* assay.

As mentioned above, compound **11h** has better activity than **11b**, and could serve as a new starting point for further optimisation. Since the aniline group is a known functional group for carcinogenicity, we decided to incorporate a nitrogen atom on the phenyl ring to mitigate such liability. Analog **11i** was designed and synthesised for head-to-head comparison in biological testing. Notably, **11i** exhibited more than 6-fold potency enhancement compared to **11h** in anti-proliferative activities against A549 cells. To determine which portion of **11i** contributed more to the activity, we designed two analogs **11j** and **15**. The absence of NH_2_ on pyridyl ring (i.e. **11j**) tenuate the activity significantly (17.56 µM *vs* 1.67 µM to H1975 cell lines, 2.5 µM *vs* <1 µM to A549 cell lines, 5.71 *vs* 1.37 µM to H460 cell lines) compared to **11i**, while the replacement of *N*-hydroxyl group with *N*-cyclopropyl group (i.e. **15**) significantly reduce the anticancer activities in both H1975 (IC_50_ > 30 µM) and H460 cells lines (IC_50_ > 30 µM). These results pointed to a conclusion that both *N*-hydroxyl carboximate moiety and NH_2_ group on pyridyl ring were very important contributors for good biological activities of **11i**.

Another important factor for biological activity could be the C−C double bond between *N*-hydroxyl carboximate and the pyridyl ring. This is supported by the biological data of **11i** and **11a**. Compound **11a**, a close analog of **11i** without a C−C double bond next to *N*-hydroxyl carboximate moiety, exhibited more than 15-fold activity loss compared to compound **11i**.

In summary, compound **11i** possessed several important elements including *N*-hydroxyl carboximate, C−C double bond, and 2-pyridyl amine group, all contributing to its potent biological activity. This compound also exhibited potent anti-proliferative activities against several tumour cell lines, including H1299 (lung cancer cells), U87MG (malignant glioma cells), MV4-11 (hematogical cells), and SU-DHL-2 (lymphoma cells) (Table 2). Thus, compound **11i** progressed to further preliminary mechanistic and *in vivo* studies.

**Table 2.** IC_50_ values of the representative compound **11i** against other tumour cell lines.

	IC_50_ (µM)^a^			
Compd.	H1299	U87MG	MV4-11	SU-DHL-2
**11i**	1.14 ± 0.15	9.34 ± 0.77	0.86 ± 0.77	2.73 ± 0.31
**1**	>100	>100	>100	>100
**SAHA**	7.08 ± 0.85	2.56 ± 0.43	<0.37	1.84 ± 0.12

^a^Data represent the mean value from at least three independent experiments.

#### 2.3.2. *In vitro* induced cell apoptosis

**Figure 5.**
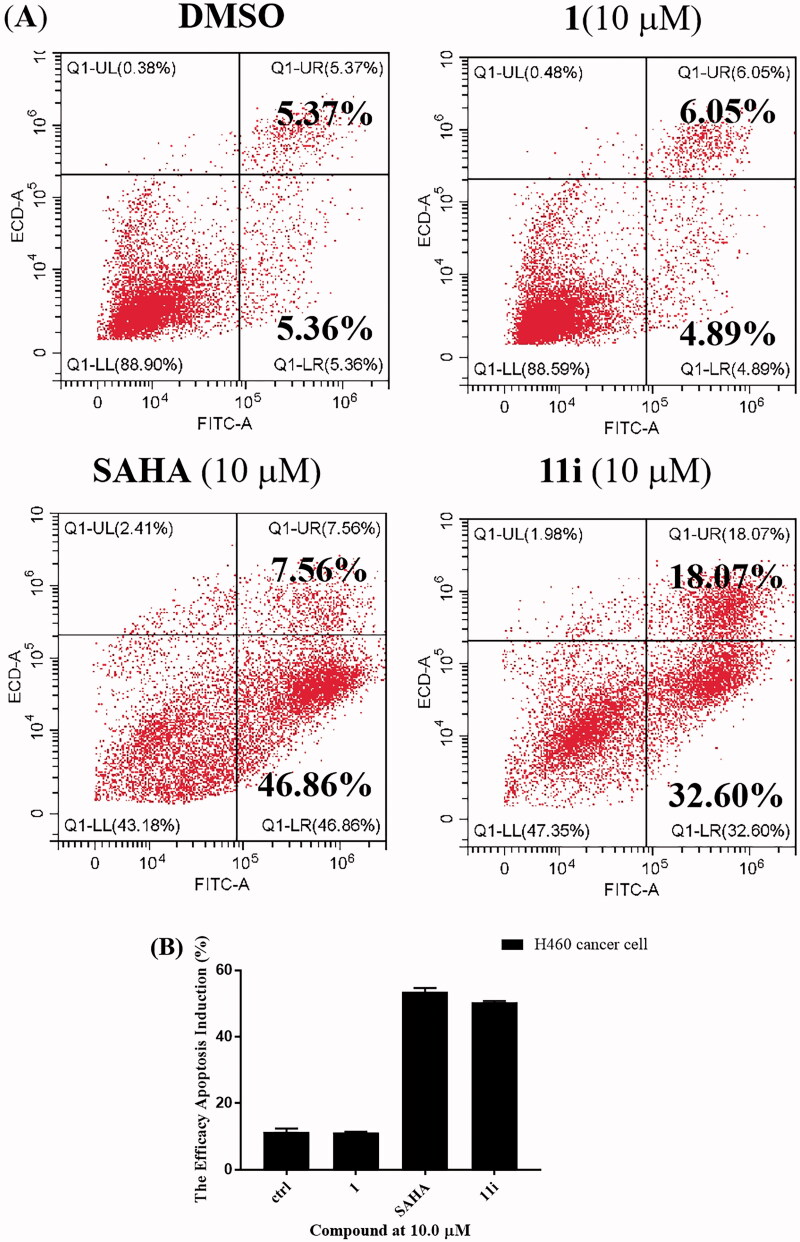
(A) Cell apoptosis is induced by compound **11i**. H460 cells were incubated with the 10 µM concentrations of **11i** for 72 h. Cells treated with DMSO were used for comparison and cells treated with **1** and **SAHA** were used for positive control. Data were represented as mean standard deviation from three independent experiments; (B) Ability of compounds **1**, **SAHA**, and **11i** to induce apoptosis in H460 cells after 72 h of treatment.

To investigate the effect of the selected representative compound on the induction of apoptosis, compound **11i** was evaluated by annexin VFTIC/propidium iodide (PI) assay. H460 cells were incubated with vehicle alone or tested compounds at 10 μM for 72 h. As shown in Figure 5, compound **11i** could significantly induce H460 cell apoptosis. The percentage of apoptotic cells for compound **11i** at the concentration of 10 μM is greater than 50%, which was obviously higher than lead compound *β*-elemene **1** (10.94% apoptotic cells at 10 μM, *p* < 0.05).

#### 2.3.3. *In vivo* anti-tumour activities against H460 xenografts

**Figure 6.**
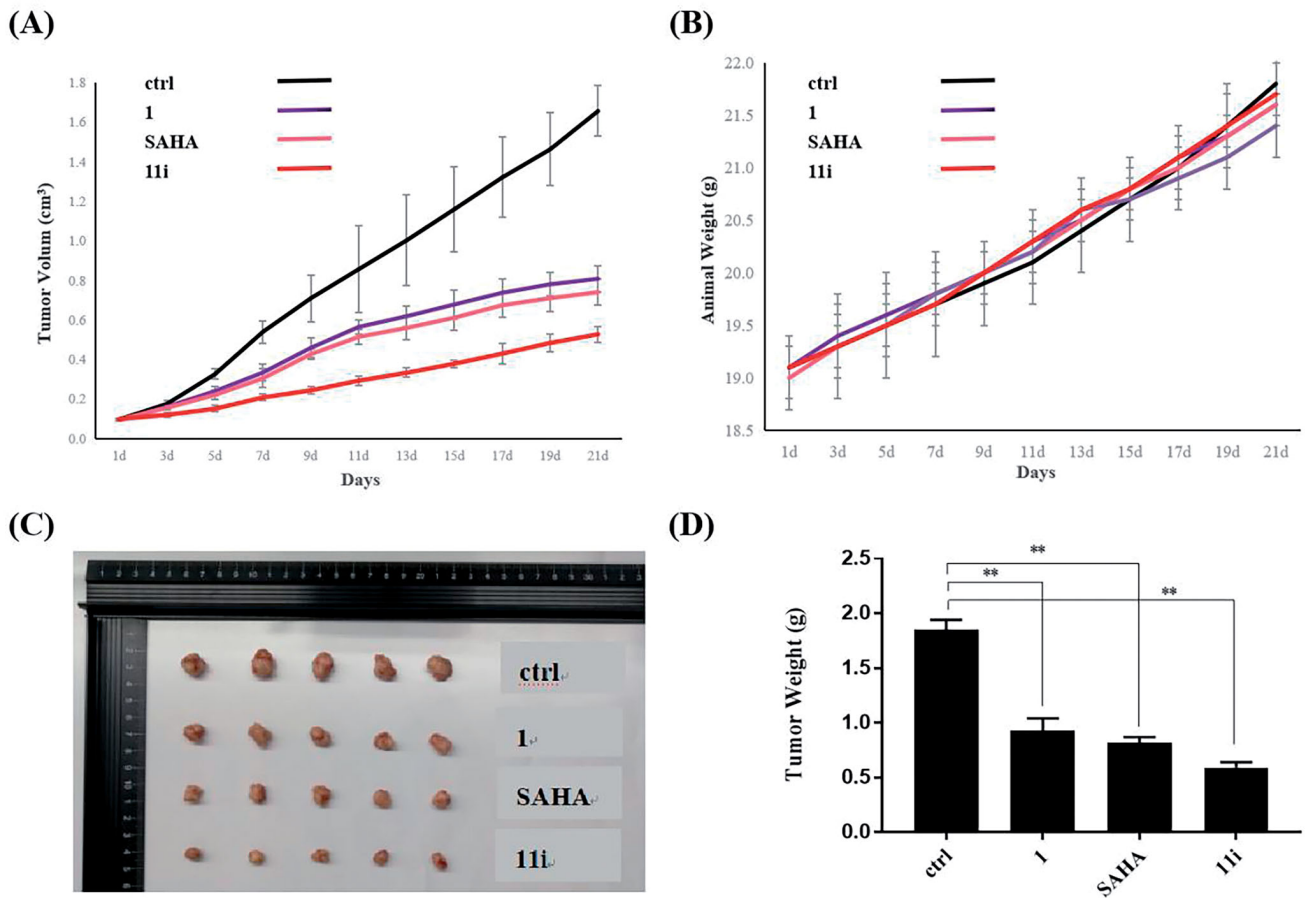
Tumour growth inhibition of compound **11i** in H460 xenograft mice model. (A) The efficacy of compound **11i** in the H460 xenograft model. (B) Average body weights for **1**, **SAHA**, **11i** and vehicle-treated mice groups. (C) Photo of dissected H460 tumour tissues. (D) Tumour weight of dissected H460 tumour tissues. Single asterisks indicate *p* < 0.05, double asterisks indicate *p* < 0.01, and triple asterisks indicate *p* < 0.001 versus the control group.

To investigate the *in vivo* anti-tumour effects, xenograft model of H460 was set up. First, compound **11i** was tested in the H460 xenograft model. H460 cells (1 × 10^6^) were implanted on right flanks subcutaneously in female nude mice. When the implanted tumour reached a volume of 80–100 mm^3^, the animals were randomly divided into groups of 4 and compound **11i** was administered intraperitoneally at 60 mg/kg once a day for 21 consecutive days. Compounds **1** and **SAHA** were used as the positive controls. As shown in Figure 6(A,C,D) and Table 3, treatment with compound **11i** caused significantly reduction in tumour growth, which showed higher tumour growth inhibition (TGI) values (68.3%) and lower treatment-to-control (T/C) values (31.18%) than positive drugs **1** (*β*-Elemene, (TGI = 50.1%; T/C = 50.25%)) and SAHA (TGI = 55.9%; T/C = 45.28%) in the xenograft H460 model. Moreover, compound **11i** was observed to be well tolerated during the test and no significant loss of body weight was observed (Figure 6B), indicating that its toxicity is low.

**Table 3.** *In vivo* anti-tumour efficacy in the H460 xenograft model

compound	administration	TGI (%)
**11i**	60 mg/(kg day), IP	68.3%
**1**	60 mg/(kg day), IP	50.1%
**SAHA**	60 mg/(kg day), IP	55.9%

#### 2.3.4. The solubility and solubility levels of the target compounds

Accelrys Discovery Studio (DS) software was used to assess the solubility level of all final products. It’s not a surprise that the solubility of many target compounds (*i.e.* **11b**–**11d**, **11f**, **17** and **15**) was as poor as that of *β*-elemene due to the highly lipophilicity of the parent. But it’s encouraging that compounds **11a** and **11h**–**11j** have slightly improved solubility compared to *β*-elemene (Table 4). As expected, compounds **11h**, **11i** and **15** exhibited much improvement of solubility level when they were in their hydrochloride forms (Table 4). Among all compounds examined in the DS software, compound **11i** is the best in term of solubility level whether it is in the free state or in its HCl salt form.

**Table 4.** The solubility and solubility levels of the target compounds of predicted by DS software.

Compound	ADMET Solubility	ADMET Solubility Level	ADMET Solubility (·HCl)	ADMET Solubility Level (·HCl)
**11a**	−4.050 ↑	2	—	—
**11b**	−5.666	2	—	—
**11c**	−5.676	2	—	—
**11d**	−5.685	2	—	—
**11e**	−5.077 ↑	2	—	—
**11f**	−6.307	1	—	—
**17**	−6.886	1	—	—
**11h**	−4.484 ↑	2	−3.419 ↑	3 ↑
**11i**	−4.025 ↑	2	−3.224 ↑	3 ↑
**11j**	−4.503 ↑	2	—	—
**15**	−5.393	2	−4.328 ↑	2
**1**	−5.295	2	—	—

Note: smaller values indicate lower solubility.

## 3. Conclusion

Structure modifications of natural products, especially those with interesting biological activity profiles such as *β*-elemene have been one of the hot areas in modern drug discovery. We reported herein a novel class of *N*-alkyl-*N*-hydroxyl carboximate derivatives of *β*-elemene with attractive anticancer activities both in *vitro* and *in vivo*. The representative compound **11i** not only exhibited marked anti-tumour effects against 6 tumour cell lines, but also possessed potent proapoptotic activity. In *in vivo* study, compound **11i** showed strong anti-tumour efficacy in the H460 xenograft mice model without observable toxicity. Although the *β*-elemene analogs described in this article exhibited much improved anti-tumour activity, their precise biological targets are yet to be uncovered. Nevertheless, this study expanded the structure scope of *β*-elemene modifications beyond those reported and provided further optimisation for medicinal research around natural products such as *β*-elemene.

## 4. Experimental section

### 4.1. Chemistry

#### 4.1.1. General information

All of the chemicals were purchased from commercial suppliers. The melting points of the compounds were determined using Büchi B-540 capillary melting point instrument. ^1^H NMR (500 MHz) and ^13 ^C NMR (126 MHz) were recorded on a 500 MHz Bruker NMR spectroscopy using CDCl_3_, CD_3_OD or DMSO-*d*_6_ as the deuterated solvent. Chemical shifts (*δ*) were reported in parts per million (ppm) relative to residual solvent as an internal reference. Low-resolution mass spectra were recorded with Agilent 1260 Infinity II/1625. High-resolution mass spectra (HRMS) were measured on a Bruker MICR OTOF-Q II instrument or Shimadzu LCMSIT-TOF mass spectrometer using the ESI technique. High performance liquid chromatography (HPLC) was determined on a Shimadzu LC-2030Plus instrument.

##### 4.1.1.1. The synthesis of the crucial intermediate 4

To a solution of *β*-elemene (**1**, 6.01 g, 29.46 mmol) in CH_3_CO_2_H (20 mL) was added NBS (6.29 g, 35.35 mmol) at 0 °C. After addition, the mixture was stirred at room temperature for 9 h. The reaction was monitored by TLC. The mixture was then neutralised with saturated NaHCO_3_ solution and extracted with petroleum ether (3 × 50 mL). The combined organic layers were washed with water and brine, and dried over Na_2_SO_4_. The drying agent was filtered off. The filtrate was concentrated under reduced pressure, and the residue was purified *via* flash column chromatography (petroleum ether as eluent) to give 13-Br-*β*-elemene **4** (2.51 g, yield 30.1%) as a light yellow liquid. ^1^H NMR (400 MHz, CDCl_3_) δ 5.89 − 5.76 (m, 1H), 5.21 (s, 1H), 5.04 (t, *J* = 1.1 Hz, 1H), 4.97 − 4.81 (m, 3H), 4.59 (*d*t, *J* = 1.9, 0.9 Hz, 1H), 4.04 (*d*, *J* = 0.7 Hz, 2H), 2.33 − 2.17 (m, 1H), 2.06 (*dd*, *J* = 12.6, 3.5 Hz, 1H), 1.74 − 1.70 (m, 3H), 1.69 − 1.39 (m, 6H), 1.01 (s, 3H).

##### 4.1.1.2. General procedure for the synthesis of intermediate 5a–5j

The solution of H_2_N−OTHP (6.65 mmol), DIPEA (8.31 mmol), EDCI (14.40 mmol), HOBt (7.20 mmol) and the corresponding acids **7** (5.54 mmol) in DMF (20 mL) was stirred at room temperature for 6 h. The reaction was monitored by TLC. Upon completion, the mixture was quenched with water and extracted with ethyl acetate (3 × 100 mL). The combined organic layers were washed with water and brine and dried over Na_2_SO_4_. The drying agent was filtered off. The filtrate was concentrated under reduced pressure and the residue was purified *via* flash column chromatography (dichloromethane/methanol 9:1, v/v) to give compounds **5**.

###### 4.1.1.2.1. 6-Amino-N-((tetrahydro-2H-pyran-2-yl)oxy)nicotinamide (5a)

White solid, yield 88.8%. ^1^H NMR (400 MHz, CD_3_OD) δ 8.36 (d, *J* = 2.4 Hz, 1H), 7.82 (dd, *J* = 8.8, 2.4 Hz, 1H), 6.57 (d, *J* = 8.8 Hz, 1H), 5.02 − 4.99 (m, 1H), 4.11 (td, *J* = 10.9, 3.1 Hz, 1H), 3.69 − 3.57 (m, 1H), 1.96 − 1.56 (m, 6H).

###### 4.1.1.2.2. (E)-N-((Tetrahydro-2H-pyran-2-yl)oxy)-3-(p-tolyl)acrylamide (5b)

White solid, yield 84.4%. ^1^H NMR (500 MHz, CDCl_3_) δ 8.45 (s, 1H), 7.71 (d, *J* = 15.6 Hz, 1H), 7.41 (d, *J* = 7.8 Hz, 2H), 7.18 (d, *J* = 7.8 Hz, 2H), 6.41 (s, 1H), 5.01 (s, 1H), 3.98 (t, *J* = 9.6 Hz, 1H), 3.67 (dtd, *J* = 11.3, 4.2, 1.8 Hz, 1H), 2.37 (s, 3H), 1.93 − 1.79 (m, 3H), 1.72 − 1.62 (m, 2H), 1.59 − 1.52 (m, 1H). LCMS *m/z* [M + H]^+^: 262.0.

###### 4.1.1.2.3. (E)-N-((Tetrahydro-2H-pyran-2-yl)oxy)-3-(m-tolyl)acrylamide (5c)

White solid, yield 70.5%. ^1^H NMR (500 MHz, CDCl_3_) δ 9.00 (s, 1H), 7.71 (d, *J* = 15.7 Hz, 1H), 7.31 (d, *J* = 6.4 Hz, 2H), 7.24 (t, *J* = 7.9 Hz, 1H), 7.17 (d, *J* = 7.5 Hz, 1H), 6.42 (s, 1H), 5.03 (s, 1H), 4.00 (t, *J* = 9.1 Hz, 1H), 3.66 (ddt, *J* = 9.6, 5.5, 2.9 Hz, 1H), 2.34 (s, 3H), 1.85 (dq, *J* = 12.7, 4.7, 4.1 Hz, 3H), 1.71 − 1.55 (m, 3H). LCMS *m/z* [M + H]^+^: 262.0.

###### 4.1.1.2.4. (E)-N-((Tetrahydro-2H-pyran-2-yl)oxy)-3-(o-tolyl)acrylamide (5d)

White solid, yield 83.8%. ^1^H NMR (500 MHz, CDCl_3_) δ 8.01 (d, *J* = 15.8 Hz, 1H), 7.50 (d, *J* = 7.3 Hz, 1H), 7.40 − 7.08 (m, 4H), 6.33 (s, 1H), 5.03 (s, 1H), 3.99 (t, *J* = 9.3 Hz, 1H), 3.66 (dtd, *J* = 11.3, 4.1, 1.8 Hz, 1H), 2.42 (s, 3H), 1.92 − 1.78 (m, 3H), 1.73 − 1.55 (m, 3H).

###### 4.1.1.2.5. (E)-3–(4-Cyanophenyl)-N-((tetrahydro-2H-pyran-2-yl)oxy)acrylamide (5e)

Yellow solid, yield 85.6%. ^1^H NMR (500 MHz, CDCl_3_) δ 8.96 (s, 1H), 7.86 − 7.39 (m, 5H), 6.66 − 6.34 (m, 1H), 5.04 (s, 1H), 3.98 (q, *J* = 10.6 Hz, 1H), 3.72 − 3.62 (m, 1H), 1.86 (qt, *J* = 10.6, 7.6, 3.5 Hz, 3H), 1.64 (ddd, *J* = 32.1, 10.6, 6.9 Hz, 3H).

###### 4.1.1.2.6. (E)-N-((Tetrahydro-2H-pyran-2-yl)oxy)-3–(4-(trifluoromethyl)phenyl)acrylamide (5f)

White solid, yield 91.3%. ^1^H NMR (500 MHz, CDCl_3_) δ 8.89 (s, 1H), 7.75 (d, *J* = 15.8 Hz, 1H), 7.62 (s, 4H), 6.67 − 6.29 (m, 1H), 5.03 (s, 1H), 3.98 (d, *J* = 11.8 Hz, 1H), 3.76 − 3.60 (m, 1H), 2.01 − 1.47 (m, 6H).

###### 4.1.1.2.7. (E)-3–(4-Hydroxyphenyl)-N-((tetrahydro-2H-pyran-2-yl)oxy)acrylamide (5g)

White solid, yield 67.4%. ^1^H NMR (400 MHz, DMSO-*d*_6_) δ 11.12 (s, 1H), 9.91 (s, 1H), 7.40 (dd, *J* = 12.0, 7.2 Hz, 3H), 6.79 (d, *J* = 8.6 Hz, 2H), 6.28 (d, *J* = 15.8 Hz, 1H), 4.89 (s, 1H), 4.02 − 3.87 (m, 1H), 3.59 − 3.47 (m, 1H), 1.84 − 1.38 (m, 6H). LCMS *m/z* [M + Na]^+^: 286.0.

###### 4.1.1.2.8. (E)-3–(4-Aminophenyl)-N-((tetrahydro-2H-pyran-2-yl)oxy)acrylamide (5h)

Yellow solid, yield: 65.0%. ^1^H NMR (500 MHz, CDCl_3_) δ 8.43 (s, 1H), 7.65 (d, *J* = 15.6 Hz, 1H), 7.34 (d, *J* = 8.2 Hz, 2H), 6.72 − 6.58 (m, 2H), 6.26 (s, 1H), 4.99 (s, 1H), 4.17 − 3.73 (m, 3H), 3.66 (ddt, *J* = 9.4, 5.2, 2.7 Hz, 1H), 1.93 − 1.79 (m, 3H), 1.74 − 1.61 (m, 3H).

###### 4.1.1.2.9. (E)-3–(6-Aminopyridin-3-yl)-N-((tetrahydro-2H-pyran-2-yl)oxy)acrylamide (5i)

Pale yellow solid, yield 71.1%. ^1^H NMR (400 MHz, CDCl_3_) δ 9.29 (s, 1H), 8.19 (s, 1H), 7.60 (d, *J* = 14.7 Hz, 2H), 6.49 (d, *J* = 8.6 Hz, 1H), 6.23 (s, 1H), 4.96 (d, *J* = 41.5 Hz, 3H), 3.99 (t, *J* = 10.2 Hz, 1H), 3.66 (dd, *J* = 10.9, 5.5 Hz, 1H), 1.96 − 1.77 (m, 4H), 1.68 − 1.62 (m, 1H), 1.60 − 1.54 (m, 1H). LCMS *m/z* [M + H]^+^: 264.4.

###### 4.1.1.2.10. (E)-3-(Pyridin-3-yl)-N-((tetrahydro-2H-pyran-2-yl)oxy)acrylamide (5j)

White solid, yield: 66.6%. ^1^H NMR (500 MHz, CDCl_3_) δ 9.22 (s, 1H), 8.77 (s, 1H), 8.59 (s, 1H), 7.92 − 7.60 (m, 2H), 7.32 (dd, *J* = 7.9, 4.8 Hz, 1H), 6.49 (s, 1H), 5.06 (s, 1H), 3.99 (t, *J* = 9.5 Hz, 1H), 3.66 (dtd, *J* = 11.2, 4.2, 2.0 Hz, 1H), 1.96 − 1.82 (m, 3H), 1.74 − 1.56 (m, 3H). LCMS *m/z* [M + H]^+^:249.0.

##### 4.1.1.3. General procedure for the synthesis of intermediate 9a–9f and 9h–9j

The solution of the 13-Br-*β*-elemene **4** (1.67 mmol), the intermediate **5** (1.84 mmol) and Cs_2_CO_3_ (2.51 mmol) in DMF (5 mL) was stirred at 60 °C for 10 h. The reaction was monitored by TLC. Upon completion, the mixture was diluted in H_2_O (30 mL) and was extracted three times with ethyl acetate (3 × 30 mL). The combined organic layers were washed with water and brine, and dried over Na_2_SO_4_. The drying agent was filtered off. The filtrate was concentrated under reduced pressure and the residue was purified *via* flash column chromatography (petroleum ether/ethyl acetate 2:3, v/v) to give intermediates **9a**–**9f** and **9h**–**9j**.

###### 4.1.1.3.1. 6-Amino-N-(2-((1R,3S,4S)-4-methyl-3-(prop-1-en-2-yl)-4-vinylcyclohexyl)allyl)-N-((tetrahydro-2H-pyran-2-yl)oxy)nicotinamide (9a)

Colourless liquid, yield 31.0%. ^1^H NMR (400 MHz, CDCl_3_) δ 8.52 (d, *J* = 2.2 Hz, 1H), 7.83 (dd, *J* = 8.6, 2.3 Hz, 1H), 6.48 (d, *J* = 8.6 Hz, 1H), 5.80 (dd, *J* = 17.8, 10.5 Hz, 1H), 5.09 − 4.71 (m, 8H), 4.58 (dd, *J* = 6.9, 2.0 Hz, 1H), 4.32 (dd, *J* = 16.4, 13.3 Hz, 1H), 3.79 (tdd, *J* = 10.9, 6.8, 2.8 Hz, 1H), 3.58 − 3.47 (m, 1H), 2.08 − 1.94 (m, 2H), 1.81 − 1.35 (m, 15H), 1.00 (s, 3H). LCMS *m/z* [M + H]^+^: 440.2.

###### 4.1.1.3.2. (E)-N-(2-((1R,3S,4S)-4-Methyl-3-(prop-1-en-2-yl)-4-vinylcyclohexyl)allyl)-N-((tetrahydro-2H-pyran-2-yl)oxy)-3-(p-tolyl)acrylamide (9b)

Pale yellow liquid, yield 20.3%. ^1^H NMR (500 MHz, CDCl_3_) δ 7.71 (d, *J* = 15.8 Hz, 1H), 7.44 (d, *J* = 7.9 Hz, 2H), 7.18 (d, *J* = 7.8 Hz, 2H), 7.03 (d, *J* = 15.7 Hz, 1H), 5.81 (dd, *J* = 17.4, 10.9 Hz, 1H), 5.04 − 4.87 (m, 5H), 4.85 − 4.70 (m, 2H), 4.58 (d, *J* = 3.8 Hz, 1H), 4.30 (dd, *J* = 22.6, 16.5 Hz, 1H), 4.05 − 3.95 (m, 1H), 3.59 (m, 1H), 2.37 (s, 3H), 2.05 − 1.96 (m, 2H), 1.90 − 1.72 (m, 5H), 1.69 − 1.41 (m, 10H), 1.00 (s, 3H). LCMS *m/z* [M + H]^+^: 464.5.

###### 4.1.1.3.3. (E)-N-(2-((1R,3S,4S)-4-Methyl-3-(prop-1-en-2-yl)-4-vinylcyclohexyl)allyl)-N-((tetrahydro-2H-pyran-2-yl)oxy)-3-(m-tolyl)acrylamide (9c)

Colourless liquid, yield 15.4%. ^1^H NMR (500 MHz, CDCl_3_) δ 7.70 (d, *J* = 15.8 Hz, 1H), 7.38 − 7.31 (m, 2H), 7.27 (t, *J* = 7.6 Hz, 1H), 7.18 (d, *J* = 7.4 Hz, 1H), 7.06 (d, *J* = 15.5 Hz, 1H), 5.81 (dd, *J* = 17.4, 10.9 Hz, 1H), 5.04 − 4.86 (m, 5H), 4.84 − 4.69 (m, 2H), 4.59 (dd, *J* = 4.9, 2.0 Hz, 1H), 4.30 (dd, *J* = 22.4, 16.5 Hz, 1H), 4.04 − 3.95 (m, 1H), 3.62 − 3.56 (m, 1H), 2.37 (s, 3H), 2.06 − 1.94 (m, 2H), 1.89 − 1.72 (m, 5H), 1.69 − 1.53 (m, 7H), 1.53 − 1.39 (m, 3H), 1.01 (s, 3H). LCMS *m/z* [M + H]^+^: 464.3.

###### 4.1.1.3.4. (E)-N-(2-((1R,3S,4S)-4-Methyl-3-(prop-1-en-2-yl)-4-vinylcyclohexyl)allyl)-N-((tetrahydro-2H-pyran-2-yl)oxy)-3-(o-tolyl)acrylamide (9d)

Colourless liquid, yield 24.5%. ^1^H NMR (500 MHz, CDCl_3_) δ 8.01 (d, *J* = 15.7 Hz, 1H), 7.56 (d, *J* = 7.3 Hz, 1H), 7.28 − 7.24 (m, 1H), 7.21 (t, *J* = 6.8 Hz, 2H), 7.00 (d, *J* = 16.0 Hz, 1H), 5.81 (dd, *J* = 17.4, 10.9 Hz, 1H), 5.07 − 4.86 (m, 5H), 4.84 − 4.68 (m, 2H), 4.60 − 4.57 (m, 1H), 4.30 (dd, *J* = 21.2, 16.5 Hz, 1H), 4.05 − 3.95 (m, 1H), 3.59 (ddt, *J* = 12.9, 6.7, 3.1 Hz, 1H), 2.45 (s, 3H), 2.05 − 1.95 (m, 2H), 1.91 − 1.71 (m, 5H), 1.69 − 1.55 (m, 7H), 1.54 − 1.41 (m, 3H), 1.01 (s, 3H).

###### 4.1.1.3.5. (E)-3–(4-Cyanophenyl)-N-(2-((1R,3S,4S)-4-methyl-3-(prop-1-en-2-yl)-4-vinylcyclohexyl)allyl)-N-((tetrahydro-2H-pyran-2-yl)oxy)acrylamide (9e)

Pale yellow liquid, yield 17.3%. ^1^H NMR (500 MHz, CDCl_3_) δ 7.78 − 7.56 (m, 5H), 7.21 (d, *J* = 15.2 Hz, 1H), 5.80 (dd, *J* = 17.3, 11.0 Hz, 1H), 5.09 − 4.78 (m, 6H), 4.75 − 4.56 (m, 2H), 4.35 (dd, *J* = 23.1, 16.4 Hz, 1H), 4.00 (dt, *J* = 11.1, 5.1 Hz, 1H), 3.58 (dd, *J* = 11.9, 5.6 Hz, 1H), 2.07 − 1.92 (m, 2H), 1.90 − 1.60 (m, 12H), 1.54 − 1.42 (m, 3H), 1.01 (s, 3H). LCMS *m/z* [M + H]^+^: 475.2.

###### 4.1.1.3.6. (E)-N-(2-((1R,3S,4S)-4-Methyl-3-(prop-1-en-2-yl)-4-vinylcyclohexyl)allyl)-N-((tetrahydro-2H-pyran-2-yl)oxy)-3–(4-(trifluoromethyl)phenyl)acrylamide (9f)

Pale yellow liquid, yield 14.4%. ^1^H NMR (500 MHz, CDCl_3_) δ 7.72 (d, *J* = 15.8 Hz, 1H), 7.63 (s, 4H), 7.18 (d, *J* = 12.3 Hz, 1H), 5.81 (dd, *J* = 17.3, 11.0 Hz, 1H), 5.06 − 4.79 (m, 6H), 4.78 − 4.56 (m, 2H), 4.34 (dd, *J* = 23.1, 16.4 Hz, 1H), 4.03 − 3.97 (m, 1H), 3.59 (dq, *J* = 9.3, 4.1, 3.0 Hz, 1H), 2.08 − 1.93 (m, 2H), 1.90 − 1.59 (m, 12H), 1.53 − 1.42 (m, 3H), 1.01 (s, 3H).

###### 4.1.1.3.7. (E)-3–(4-Aminophenyl)-N-(2-((1R,3S,4S)-4-methyl-3-(prop-1-en-2-yl)-4-vinylcyclohexyl)allyl)-N-((tetrahydro-2H-pyran-2-yl)oxy)acrylamide (9h)

Pale yellow solid, yield 20.2%. ^1^H NMR (500 MHz, CDCl_3_) δ 7.65 (d, *J* = 15.7 Hz, 1H), 7.39 − 7.34 (m, 2H), 6.91 − 6.83 (m, 1H), 6.68 − 6.63 (m, 2H), 5.80 (dd, *J* = 17.4, 10.9 Hz, 1H), 5.03 − 4.86 (m, 5H), 4.84 − 4.70 (m, 2H), 4.61 − 4.56 (m, 1H), 4.27 (dd, *J* = 22.5, 16.5 Hz, 1H), 4.00 (tt, *J* = 8.3, 1.8 Hz, 1H), 3.91 (s, 2H), 3.62 − 3.56 (m, 1H), 2.00 (tdd, *J* = 14.7, 12.1, 8.3 Hz, 2H), 1.89 − 1.71 (m, 5H), 1.67 − 1.54 (m, 7H), 1.51 − 1.41 (m, 3H), 1.00 (s, 3H). LCMS *m/z* [M + H]^+^: 487.0.

###### 4.1.1.3.8. (E)-3–(6-Aminopyridin-3-yl)-N-(2-((1R,3S,4S)-4-methyl-3-(prop-1-en-2-yl)-4-vinylcyclohexyl)allyl)-N-((tetrahydro-2H-pyran-2-yl)oxy)acrylamide (9i)

Pale yellow liquid, yield: 20.1%. ^1^H NMR (400 MHz, CDCl_3_) δ 8.17 (d, *J* = 2.3 Hz, 1H), 7.62 − 7.49 (m, 2H), 6.85 (d, *J* = 15.7 Hz, 1H), 6.44 (d, *J* = 8.6 Hz, 1H), 5.74 (dd, *J* = 17.4, 10.9 Hz, 1H), 4.96 − 4.79 (m, 5H), 4.77 − 4.59 (m, 4H), 4.51 (t, *J* = 2.9 Hz, 1H), 4.22 (dd, *J* = 19.6, 16.5 Hz, 1H), 3.98 − 3.87 (m, 1H), 3.52 (dd, *J* = 11.5, 6.5 Hz, 1H), 1.97 − 1.86 (m, 2H), 1.82 − 1.64 (m, 4H), 1.63 (d, *J* = 3.5 Hz, 3H), 1.59 − 1.46 (m, 5H), 1.46 − 1.33 (m, 3H), 0.93 (s, 3H). LCMS *m/z* [M + H]^+^: 466.8.

###### 4.1.1.3.9. (E)-N-(2-((1R,3S,4S)-4-Methyl-3-(prop-1-en-2-yl)-4-vinylcyclohexyl)allyl)-3-(pyridin-3-yl)-N-((tetrahydro-2H-pyran-2-yl)oxy)acrylamide (9j)

Colourless liquid, yield 16.0%. ^1^H NMR (500 MHz, CDCl_3_) δ 8.80 (s, 1H), 8.58 (d, *J* = 3.8 Hz, 1H), 7.82 (dt, *J* = 8.0, 2.0 Hz, 1H), 7.70 (d, *J* = 15.9 Hz, 1H), 7.33 (dd, *J* = 7.9, 4.8 Hz, 1H), 7.21 (d, *J* = 15.8 Hz, 1H), 5.81 (dd, *J* = 17.3, 11.0 Hz, 1H), 5.03 − 4.87 (m, 5H), 4.82 (q, *J* = 1.5 Hz, 1H), 4.69 (dd, *J* = 33.3, 16.3 Hz, 1H), 4.58 (s, 1H), 4.34 (dd, *J* = 23.1, 16.4 Hz, 1H), 4.00 (dt, *J* = 11.2, 4.8 Hz, 1H), 3.63 − 3.54 (m, 1H), 2.04 − 1.94 (m, 2H), 1.91 − 1.72 (m, 5H), 1.69 − 1.55 (m, 7H), 1.53 − 1.41 (m, 3H), 1.01 (s, 3H).

##### 4.1.1.4. General procedure for the synthesis of the target products 11a–11f and 11h–11i

To a solution of **9** (0.19 mmol) in methanol (3 mL) was added TsOH·H_2_O (0.57 mmol) and the resulted solution was stirred at room temperature for 8 h. The reaction was monitored by TLC. Upon completion, the mixture was concentrated under reduced pressure. The residue was diluted in H_2_O (10 mL) and extracted three times with dichloromethane (3 × 10 mL). The combined organic layers were washed with water and brine and dried over Na_2_SO_4_. The drying agent was filtered off. The filtrate was concentrated under reduced pressure and the residue was purified *via* reversed-phase (C18) column chromatography (water/acetonitrile 2:3) to afford compounds **11**.

###### 4.1.1.4.1. 6-Amino-N-hydroxy-N-(2-((1R,3S,4S)-4-methyl-3-(prop-1-en-2-yl)-4-vinylcyclohexyl)allyl)nicotinamide (11a)

White solid, yield 80.3%, m.p. 90.9 − 92.7 °C. ^1^H NMR (500 MHz, DMSO-*d*_6_) δ 9.72 (s, 1H), 8.37 (d, *J* = 2.4 Hz, 1H), 7.73 (dd, *J* = 8.7, 2.4 Hz, 1H), 6.53 − 6.32 (m, 3H), 5.81 (dd, *J* = 17.8, 10.5 Hz, 1H), 5.06 − 4.83 (m, 4H), 4.78 (t, *J* = 1.9 Hz, 1H), 4.58 (d, *J* = 2.3 Hz, 1H), 4.26 (s, 2H), 2.00 (ddd, *J* = 12.0, 9.9, 3.7 Hz, 2H), 1.72 − 1.32 (m, 9H), 0.96 (s, 3H). ^13 ^C NMR (126 MHz, DMSO-*d*_6_) δ 167.52, 161.24, 150.46, 150.16, 148.93, 147.56, 138.35, 118.29, 112.71, 110.72, 110.50, 106.69, 53.33, 52.41, 41.78, 39.94, 32.86, 27.04, 25.08, 16.79. High-resolution mass spectrometry (HRMS) (electrospray ionisation (ESI)), [M + H]^+^ *m*/*z*: 356.2355. HPLC purity of 98.91%.

###### 4.1.1.4.2. (E)-N-Hydroxy-N-(2-((1R,3S,4S)-4-methyl-3-(prop-1-en-2-yl)-4-vinylcyclohexyl)allyl)-3-(p-tolyl)acrylamide (11b)

White solid, yield 70.8%, m.p. 121.6 − 123.7 °C. ^1^H NMR (500 MHz, DMSO-*d*_6_) δ 9.83 (s, 1H), 7.58 − 7.46 (m, 3H), 7.23 (d, *J* = 7.9 Hz, 3H), 5.81 (dd, *J* = 17.9, 10.5 Hz, 1H), 4.97 (s, 1H), 4.94 − 4.84 (m, 3H), 4.80 − 4.77 (m, 1H), 4.59 (d, *J* = 2.3 Hz, 1H), 4.27 (s, 2H), 2.33 (s, 3H), 1.98 (dt, *J* = 13.8, 7.1 Hz, 2H), 1.71 − 1.52 (m, 6H), 1.49 − 1.33 (m, 3H), 0.97 (s, 3H). ^13 ^C NMR (126 MHz, DMSO-*d*_6_) δ 150.47, 148.79, 147.56, 139.99, 132.69, 130.00, 128.31, 116.64, 112.72, 110.50, 52.39, 41.58, 32.79, 27.03, 25.07, 21.44, 16.78. HRMS (ESI) [M + Na]^+^ *m*/*z*: 402.2423. HPLC purity of 95.46%.

###### 4.1.1.4.3. (E)-N-Hydroxy-N-(2-((1R,3S,4S)-4-methyl-3-(prop-1-en-2-yl)-4-vinylcyclohexyl)allyl)-3-(m-tolyl)acrylamide (11c)

Colourless liquid, yield 94.0%. ^1^H NMR (500 MHz, DMSO-*d*_6_) δ 9.91 (s, 1H), 7.56 − 7.43 (m, 3H), 7.37 − 7.20 (m, 3H), 5.82 (dd, *J* = 17.9, 10.5 Hz, 1H), 4.99 (s, 1H), 4.95 − 4.85 (m, 3H), 4.80 (t, *J* = 1.9 Hz, 1H), 4.60 (s, 1H), 4.29 (s, 2H), 2.35 (s, 3H), 1.99 (dt, *J* = 13.7, 6.8 Hz, 2H), 1.75 − 1.53 (m, 6H), 1.52 − 1.34 (m, 3H), 0.98 (s, 3H). ^13 ^C NMR (126 MHz, DMSO-*d*_6_) δ 150.46, 148.76, 147.55, 141.82, 138.61, 135.36, 130.90, 129.29, 128.71, 125.64, 117.54, 112.72, 110.50, 52.38, 52.14, 41.60, 32.79, 27.04, 25.07, 21.34, 16.77. HRMS (ESI) [M + Na]^+^ *m*/*z*: 402.2416. HPLC purity of 98.74%.

###### 4.1.1.4.4. (E)-N-Hydroxy-N-(2-((1R,3S,4S)-4-methyl-3-(prop-1-en-2-yl)-4-vinylcyclohexyl)allyl)-3-(o-tolyl)acrylamide (11d)

Colourless liquid, yield 73.1%. ^1^H NMR (500 MHz, CD_3_OD) δ 7.92 (d, *J* = 15.8 Hz, 1H), 7.59 (d, *J* = 7.6 Hz, 1H), 7.26 − 7.16 (m, 4H), 5.80 (dd, *J* = 17.5, 10.8 Hz, 1H), 4.99 (d, *J* = 24.1 Hz, 2H), 4.92 − 4.76 (m, 5H), 4.58 (s, 1H), 4.35 (s, 2H), 2.41 (s, 3H), 2.01 (dp, *J* = 13.5, 6.8, 6.0 Hz, 2H), 1.74 − 1.60 (m, 6H), 1.55 − 1.39 (m, 3H), 1.00 (s, 3H). ^13 ^C NMR (126 MHz, DMSO-*d*_6_) δ 150.13, 148.09, 147.45, 140.18, 137.31, 133.87, 130.40, 129.44, 126.07, 125.89, 117.05, 111.37, 110.30, 109.03, 52.62, 52.02, 42.04, 39.79, 39.46, 32.88, 26.86, 24.00, 18.45, 15.74. HRMS (ESI) [M + H]^+^ *m*/*z*: 402.2392. HPLC purity of 99.70%.

###### 4.1.1.4.5. (E)-3–(4-Cyanophenyl)-N-hydroxy-N-(2-((1R,3S,4S)-4-methyl-3-(prop-1-en-2-yl)-4-vinylcyclohexyl)allyl)acrylamide (11e)

Yellow solid, yield 83.2%, m.p. 86.9 − 87.8 °C. ^1^H NMR (500 MHz, DMSO-*d*_6_) δ 9.99 (s, 1H), 7.87 (s, 4H), 7.59 (d, *J* = 15.9 Hz, 1H), 7.42 (d, *J* = 15.9 Hz, 1H), 5.80 (dd, *J* = 17.8, 10.5 Hz, 1H), 5.06 − 4.74 (m, 5H), 4.58 (s, 1H), 4.28 (s, 2H), 2.07 − 1.87 (m, 2H), 1.76 − 1.31 (m, 9H), 0.96 (s, 3H). ^13 ^C NMR (126 MHz, DMSO-*d*_6_) δ 165.18, 150.44, 148.55, 147.54, 140.03, 139.80, 133.24, 129.05, 121.34, 119.13, 112.72, 112.09, 110.98, 110.50, 52.38, 52.17, 41.60, 32.76, 27.03, 25.06, 16.78. HRMS (ESI) [M + Na]^+^ *m*/*z*: 413.2179. HPLC purity of 91.98%.

###### 4.1.1.4.6. (E)-N-Hydroxy-N-(2-((1R,3S,4S)-4-methyl-3-(prop-1-en-2-yl)-4-vinylcyclohexyl)allyl)-3–(4-(trifluoromethyl)phenyl)acrylamide (11f)

Yellow solid, yield 79.1%, m.p. 94.4 − 96.7 °C. ^1^H NMR (500 MHz, DMSO-*d*_6_) δ 9.96 (s, 1H), 7.91 (d, *J* = 8.1 Hz, 2H), 7.78 (d, *J* = 8.0 Hz, 2H), 7.63 (d, *J* = 16.0 Hz, 1H), 7.44 (d, *J* = 15.9 Hz, 1H), 5.82 (dd, *J* = 17.6, 10.7 Hz, 1H), 5.05 − 4.84 (m, 4H), 4.80 (s, 1H), 4.60 (s, 1H), 4.30 (s, 2H), 1.99 (dt, *J* = 11.5, 5.6 Hz, 2H), 1.75 − 1.34 (m, 9H), 0.98 (s, 3H). ^13 ^C NMR (126 MHz, DMSO-*d*_6_) δ 165.29, 150.44, 148.58, 147.53, 139.96, 139.46, 128.97, 126.19, 125.64, 120.61, 112.70, 110.95, 110.48, 52.38, 52.14, 41.60, 32.76, 27.03, 25.05, 16.76. HRMS (ESI) [M + Na]^+^ *m*/*z*: 456.2143. HPLC purity of 92.37%.

###### 4.1.1.4.7. (E)-3–(4-Aminophenyl)-N-hydroxy-N-(2-((1R,3S,4S)-4-methyl-3-(prop-1-en-2-yl)-4-vinylcyclohexyl)allyl)acrylamide (11h)

Yellow solid, yield 50.6%, m.p. 189.2 − 190.7 °C. ^1^H NMR (500 MHz, DMSO-*d*_6_) δ 9.73 (s, 1H), 7.43 − 7.32 (m, 3H), 6.96 (d, *J* = 16.1 Hz, 1H), 6.60 (d, *J* = 8.5 Hz, 2H), 5.85 (dd, *J* = 17.9, 10.4 Hz, 1H), 5.65 (d, *J* = 8.4 Hz, 1H), 5.01 − 4.87 (m, 4H), 4.85 − 4.80 (m, 1H), 4.62 (d, *J* = 2.3 Hz, 1H), 4.28 (s, 2H), 2.09 − 1.94 (m, 2H), 1.77 − 1.35 (m, 9H), 1.00 (s, 3H). ^13 ^C NMR (126 MHz, DMSO-*d*_6_) δ 155.96, 155.24, 153.88, 152.33, 147.47, 134.73, 127.54, 118.89, 118.85, 117.46, 115.79, 115.23, 57.14, 46.29, 37.54, 31.78, 29.83, 21.53. HRMS (ESI) [M + H]^+^ *m*/*z*: 381.2520. HPLC purity of 98.86%.

###### 4.1.1.4.8. (E)-3–(6-Aminopyridin-3-yl)-N-hydroxy-N-(2-((1R,3S,4S)-4-methyl-3-(prop-1-en-2-yl)-4-vinylcyclohexyl)allyl)acrylamide (11i)

White solid, yield 70.3%, m.p. 110.5 − 112.3 °C. ^1^H NMR (500 MHz, DMSO-*d*_6_) δ 9.74 (s, 1H), 8.12 (d, *J* = 2.4 Hz, 1H), 7.73 (dd, *J* = 8.7, 2.5 Hz, 1H), 7.39 (d, *J* = 15.7 Hz, 1H), 6.99 (d, *J* = 15.8 Hz, 1H), 6.46 (q, *J* = 8.0, 7.4 Hz, 3H), 5.85 − 5.75 (m, 1H), 4.99 − 4.84 (m, 4H), 4.78 (t, *J* = 1.9 Hz, 1H), 4.59 (d, *J* = 2.2 Hz, 1H), 4.26 (s, 2H), 1.97 (dt, *J* = 13.8, 6.9 Hz, 2H), 1.70 − 1.52 (m, 6H), 1.50 − 1.32 (m, 3H), 0.96 (s, 3H). ^13 ^C NMR (126 MHz, DMSO-*d*_6_) δ 161.12, 150.54, 150.48, 149.02, 147.56, 139.70, 135.30, 119.71, 112.71, 112.40, 110.49, 108.77, 52.39, 41.54, 32.79, 27.04, 25.07, 16.78. HRMS (ESI) [M + H]^+^ *m*/*z*: 382.2499. HPLC purity of 99.13%.

###### 4.1.1.4.9. (E)-N-Hydroxy-N-(2-((1R,3S,4S)-4-methyl-3-(prop-1-en-2-yl)-4-vinylcyclohexyl)allyl)-3-(pyridin-3-yl)acrylamide (11j)

Pale yellow liquid, yield 91.7%. ^1^H NMR (500 MHz, DMSO-*d*_6_) δ 9.97 (s, 1H), 8.84 (d, *J* = 2.2 Hz, 1H), 8.57 (dd, *J* = 4.8, 1.6 Hz, 1H), 8.12 (dt, *J* = 8.0, 2.0 Hz, 1H), 7.57 (d, *J* = 15.9 Hz, 1H), 7.48 − 7.36 (m, 2H), 5.81 (dd, *J* = 17.8, 10.5 Hz, 1H), 5.02 − 4.84 (m, 4H), 4.78 (s, 1H), 4.59 (s, 1H), 4.28 (s, 2H), 2.04 − 1.91 (m, 2H), 1.74 − 1.33 (m, 10H), 0.97 (s, 3H). ^13 ^C NMR (126 MHz, DMSO-*d*_6_) δ 150.81, 150.45, 149.99, 147.54, 138.36, 134.68, 131.21, 124.42, 119.81, 112.72, 110.94, 110.51, 52.39, 52.14, 41.58, 32.78, 27.03, 25.06, 16.78. HRMS (ESI) [M + H]^+^ *m*/*z*: 367.2373. HPLC purity of 97.74%.

##### 4.1.1.5. The synthesis of the compound 13

A solution of *N*-Boc-cyclopropylamine (**12**, 2.74 mmol), Cs_2_CO_3_ (2.74 mmol) and 13-Br-*β*-elemene **4** (2.28 mmol) in DMF (7 mL) was stirred at 60 °C for overnight. The reaction was monitored by TLC. The mixture was quenched with water (35 mL) at room temperature and extracted with ethyl acetate (3 × 35 mL). The combined organic layers were washed with water and brine and dried over Na_2_SO_4_. The drying agent was filtered off. The filtrate was concentrated under reduced pressure and the residue was purified *via* column chromatography (petroleum ether/ethyl acetate 4:1, v/v) to give compound **13** (738.6 mg, yield 90.1%) as a pale yellow liquid. ^1^H NMR (500 MHz, DMSO-*d*_6_) δ 5.81 (dd, *J* = 17.9, 10.5 Hz, 1H), 4.96 − 4.84 (m, 3H), 4.82 − 4.66 (m, 2H), 4.61 − 4.55 (m, 1H), 3.78 (q, *J* = 16.3 Hz, 2H), 2.07 − 1.83 (m, 2H), 1.76 − 1.31 (m, 18H), 0.97 (s, 3H), 0.68 − 0.48 (m, 4H).

##### 4.1.1.6. The synthesis of the compound 14

To a solution of the compound **13** (1.29 mmol) in methanol (1.5 mL) was added a solution of HCl in Dioxane (4 M, 6 mL) at room temperature. The mixture was stirred at room temperature for 8 h and the reaction was monitored by TLC. Upon completion, the mixture was concentrated under reduced pressure to afford compound **14**. The crude products were used in the following reaction without further purification.

##### 4.1.1.7. The synthesis of the target products 15

A solution of **7i** (0.73 mmol), DIPEA (1.83 mmol), EDCI (1.59 mmol), HOBt (0.79 mmol) and compound **14** (0.61 mmol) in DMF (3 mL) was stirred at room temperature for 6 h. The reaction was monitored by TLC. Upon completion, the mixture was quenched with water (15 mL) and extracted with ethyl acetate (3 × 15 mL). The combined organic layers were washed with water and brine and dried over Na_2_SO_4_. The drying agent was filtered off. The filtrate was concentrated under reduced pressure and the residue was purified via column chromatography (dichloromethane/methanol 19:1, v/v) to give compound **15** (124.4 mg, yield 50.3%) as a white solid, m.p. 102.8 − 104.9 °C. ^1^H NMR (500 MHz, DMSO-*d*_6_) δ 8.12 (s, 1H), 7.76 (d, *J* = 8.7 Hz, 1H), 7.37 (d, *J* = 15.5 Hz, 1H), 7.16 (d, *J* = 15.6 Hz, 1H), 6.59 − 6.35 (m, 3H), 4.98 − 4.64 (m, 5H), 4.21 − 3.93 (m, 3H), 2.76 (tt, *J* = 9.3, 4.8 Hz, 1H), 2.28 (dd, *J* = 12.3, 3.4 Hz, 1H), 1.93 (dt, *J* = 11.9, 6.3 Hz, 1H), 1.75 − 1.61 (m, 5H), 1.58 − 1.37 (m, 3H), 1.08 (s, 3H), 0.90 (s, 2H), 0.73 (s, 2H). ^13 ^C NMR (126 MHz, DMSO-*d*_6_) δ 167.37, 160.57, 149.90, 146.22, 138.81, 134.90, 119.32, 114.22, 114.06, 108.17 (d, *J* = 14.8 Hz), 69.48, 49.08, 48.53, 41.91, 40.95, 33.86, 31.35, 29.43, 26.29, 22.51, 20.20, 19.47, 8.81. HRMS (ESI) [M + Na]^+^ *m*/*z*: 428.2678. HPLC purity of 94.27%.

###### 4.1.1.8. (E)-N-(2-((1R,3S,4S)-4-Methyl-3-(prop-1-en-2-yl)-4-vinylcyclohexyl)allyl)-3–(4-((2-((1R,3S,4S)-4-methyl-3-(prop-1-en-2-yl)-4-vinylcyclohexyl)allyl)oxy)phenyl)-N-((tetrahydro-2H-pyran-2-yl)oxy)acrylamide (16)

The solution of the 13-Br-*β*-elemene **4** (1.67 mmol), the intermediate **5g** (0.83 mmol) and Cs_2_CO_3_ (1.80 mmol) in DMF (5 mL) was stirred at 60 °C for 10 h. The reaction was monitored by TLC. Upon completion, the mixture was diluted in H_2_O (30 mL) and was extracted three times with ethyl acetate (3 × 30 mL). The combined organic layers were washed with water and brine and dried over Na_2_SO_4_. The drying agent was filtered off. The filtrate was concentrated under reduced pressure and the residue was purified via flash column chromatography (petroleum ether/ethyl acetate 3:1, v/v) to give intermediate **16** (92 mg, yield 16.6%) as a pale yellow liquid. ^1^H NMR (400 MHz, CDCl_3_) δ 7.69 (d, *J* = 15.8 Hz, 1H), 7.53 − 7.43 (m, 2H), 7.03 − 6.81 (m, 3H), 5.81 (ddd, *J* = 18.5, 10.6, 6.6 Hz, 2H), 5.16 (d, *J* = 1.4 Hz, 1H), 5.08 (s, 1H), 5.03 − 4.76 (m, 8H), 4.61 − 4.52 (m, 3H), 4.36 − 4.20 (m, 1H), 4.00 (t, *J* = 9.4 Hz, 1H), 3.59 (d, *J* = 11.5 Hz, 1H), 2.20 − 1.91 (m, 4H), 1.78 − 1.61 (m, 12H), 1.56 − 1.40 (m, 6H), 1.01 (d, *J* = 7.0 Hz, 6H).

###### 4.1.1.9. (E)-N-Hydroxy-N-(2-((1R,3S,4S)-4-methyl-3-(prop-1-en-2-yl)-4-vinylcyclohexyl)allyl)-3–(4-((2-((1R,3S,4S)-4-methyl-3-(prop-1-en-2-yl)-4-vinylcyclohexyl)allyl)oxy)phenyl)acrylamide (17)

To a solution of **16** (0.13 mmol) in methanol (3 mL) was added TsOH·H_2_O (0.39 mmol) and the resulted solution was stirred at room temperature for 8 h. The reaction was monitored by TLC. Upon completion, the mixture was concentrated under reduced pressure. The residue was diluted in H_2_O (10 mL) and extracted three times with dichloromethane (3 × 10 mL). The combined organic layers were washed with water and brine and dried over Na_2_SO_4_. The drying agent was filtered off. The filtrate was concentrated under reduced pressure and the residue was purified *via* reversed-phase (C18) column chromatography (water/acetonitrile 1:9) to afford compound **17** (61.4 mg, yield 80.9%) as a colourless liquid. ^1^H NMR (500 MHz, DMSO-*d*_6_) δ 9.83 (s, 1H), 7.63 − 7.57 (m, 2H), 7.49 (d, *J* = 15.8 Hz, 1H), 7.15 (d, *J* = 16.0 Hz, 1H), 7.07 − 6.94 (m, 2H), 5.90 − 5.75 (m, 2H), 5.19 − 4.78 (m, 10H), 4.70 − 4.54 (m, 4H), 4.28 (s, 2H), 2.20 − 1.93 (m, 4H), 1.75 − 1.34 (m, 18H), 0.99 (d, *J* = 10.3 Hz, 6H). ^13 ^C NMR (126 MHz, CDCl_3_) δ 160.29, 149.96 (d, *J* = 12.4 Hz), 148.84, 147.40 (d, *J* = 7.7 Hz), 142.88, 129.55, 115.11, 112.30, 111.10, 110.85, 110.09, 70.32, 52.66 (d, *J* = 12.1 Hz), 41.46, 40.26 − 39.31 (m), 33.15, 27.08 (d, *J* = 6.9 Hz), 24.87 (d, *J* = 3.8 Hz), 16.61 (d, *J* = 2.3 Hz). HRMS (ESI) [M + H]^+^ *m*/*z*: 584.4094. HPLC purity of 97.60%.

### 4.2. Biological evaluation

#### 4.2.1. *In vitro* anti-proliferative assay

The anti-proliferative activities of the compounds were determined by CCK8 assay. 80 μL of H1975, A549, H460, H1299, U87MG, MV4-11, and SU-DHL-2 cell suspensions (5.0 × 10^4^ cell/mL) were added to a 96-well cell culture plate and incubated for 24 h at 37 °C under an atmosphere of 5% CO_2_. *β*-Elemene derivatives were dissolved in the culture medium with 0.5% DMSO at different concentrations. The cells were treated with the drug solution for another 72 h. Then 10 µL of cell counting kit-8 (CCK8) solution was added to each well and the plate was incubated for an additional 1 h. The IC_50_ values were calculated according to the dose-dependent curves. All the tests were repeated in three independent experiments.

#### 4.2.2. Apoptosis detection assay

H460 cells were seeded into 6-well plates and incubated at 37 °C for 24 h, and then treated with or without **11i** at a concentration of 10 μM for another 72 h. The cells were then harvested by trypsinization and washed twice with cold PBS. After the centrifugation and removal of the supernatants, cells were resuspended in 500 μL of a 1 × binding buffer, which was then added to 5 μL of annexin V-FITC and 10 μL of PI, and incubated at room temperature for 15 min in the dark. The stained cells were analysed by a flow cytometer.

#### 4.2.3. H460 xenograft tumour mice model

The experimental procedures and the animal use and care protocols were approved by the Committee on Ethical Use of Animals of Hangzhou Normal University. BABL/c nude female mice (certificate SCXK-2017–0005, weighing 19.0 to 19.5 g) were obtained from Shanghai Slack Laboratory Animal Co., Ltd. H460 cell suspensions were implanted subcutaneously into the right axilla region of mice. After 18 days of cell inoculation, the animals were randomly divided into 4 groups of 5 animals. At the same time, the nude mice in each group were administrated saline, *β*-elemene (60 mg/kg), SAHA (60 mg/kg) and compound **11i** (60 mg/kg) intraperitoneally for 21 consecutive days. Tumour volumes (TV) were monitored by calliper measurement of the length and width and calculated using the formula of TV = ^1^/_2_ × *a* ×* b*, where in *a* is the tumour length and *b* is the width. Tumour volumes and body weights were recorded every 2 days over the course of treatment. Mice were sacrificed on day 39 after implantation of cells and tumours were removed, photographed and weighed for analysis.

## Supplementary Material

Supplemental Material

## References

[1] Cheng H, Ge X, Zhuo S, et al. *β*-Elemene synergizes with gefitinib to inhibit stem-like phenotypes and progression of lung cancer via down-regulating EZH2. Front Pharmacol 2018;9:1413. 30555330 10.3389/fphar.2018.01413 PMC6284059

[2] Wang X, Liu Z, Sui X, et al. Elemene injection as adjunctive treatment to platinum-based chemotherapy in patients with stage III/IV non-small cell lung cancer: a meta-analysis following the PRISMA guidelines. Phytomedicine 2019;59:152787. 31005810 10.1016/j.phymed.2018.12.010

[3] Barrero AF, Herrador MM, Quílez del Moral JF, et al. Efficient synthesis of the anticancer beta-elemene and other bioactive elemanes from sustainable germacrone. Org Biomol Chem 2011;9:1118–25. 21186393 10.1039/c0ob00467g

[4] Chen SL, You J, Wang GJ. Supercritical fluid extraction of *β*-elemene under lower pressure. Chin J Chromatogr 2001;19:179–181. 12541670

[5] Li CL, Chang L, Guo L, et al. *β*-elemene induces caspase-dependent apoptosis in human glioma cells in vitro through the upregulation of Bax and Fas/FasL and downregulation of Bcl-2. Asian Pac J Cancer Prev 2014;15:10407–12. 25556484 10.7314/apjcp.2014.15.23.10407

[6] Liu J, Zhang Y, Qu J, et al. *β*-Elemene-induced autophagy protects human gastric cancer cells from undergoing apoptosis. BMC Cancer 2011;11:183. 21595977 10.1186/1471-2407-11-183 PMC3115914

[7] Min G, Ying L, Jian Z, et al. Beta-elemene inhibits cell proliferation by regulating the expression and activity of topoisomerases I and II[alpha] in human hepatocarcinoma HepG-2 cells. Biomed Res Int 2015;2015:153987. 26221582 10.1155/2015/153987 PMC4499621

[8] Wang G, Li X, Huang F, et al. Antitumor effect of beta-elemene in non-small-cell lung cancer cells is mediated via induction of cell cycle arrest and apoptotic cell death. Cell Mol Life Sci 2005;62:881–93. 15868411 10.1007/s00018-005-5017-3 PMC11924554

[9] Tan G, Wang Z, Che L, et al. Immunotherapeutic effects on murine pancreatic carcinoma by beta-elemene combined with dendritic cells modified with genes encoding interleukin-23. Front Med China 2007;1:41–5. 24557615 10.1007/s11684-007-0008-4

[10] Bai R, Jie X, Salgado E, et al. Rational design, synthesis and biological evaluation of novel derivatives based on in vivo metabolism of natural product *β*-elemene. Lett Drug Des Discov 2018;15:905–12.

[11] Chen J, Duan W, Bai R, et al. Design, synthesis and antioxidant activity evaluation of novel *β*-elemene derivatives. Bioorg Med Chem Lett 2014;24:3407–11. 24928401 10.1016/j.bmcl.2014.05.078

[12] Liu G, Kong Z, Shen Y. Synthesis, characterization, and in vitro anti-proliferative activity of novel *β*-elemene monosubstituted derivatives. Med Chem Res 2013;22:3536–40.

[13] Ren Y, Sun Y, Cheng K, et al. Synthesis and radiolabelling of Re(CO)_3_-*β*-elemene derivatives as potential therapeutic radiopharmaceuticals. J Label Compd Radiopharm 2009;52:139–45.

[14] Sun Y, Liu G, Zhang Y, et al. Synthesis and in vitro anti-proliferative activity of *β*-elemene monosubstituted derivatives in HeLa cells mediated through arrest of cell cycle at the G1 phase. Bioorg Med Chem 2009;17:1118–24. 19128976 10.1016/j.bmc.2008.12.040

[15] Yu Z, Wang R, Xu L, et al. *β*-Elemene piperazine derivatives induce apoptosis in human leukemia cells through downregulation of c-FLIP and generation of ROS. PLoS One 2011;6:e15843. 21283566 10.1371/journal.pone.0015843 PMC3026787

[16] Yu Z, Wu F, Chen L, et al. ETME, a novel *β*-elemene derivative, synergizes with arsenic trioxide in inducing apoptosis and cell cycle arrest in hepatocarcinoma cells via a p53-dependent pathway. Acta Pharm Sin B 2014;4:424–9. 26579413 10.1016/j.apsb.2014.10.001 PMC4629106

[17] He X, Zhuo X-T, Gao Y, et al. *β*-Elemene derivatives produced from SeO_2_-mediated oxidation reaction. Royal Soc Open Sci 2020;7:200038. 10.1098/rsos.200038 PMC7277271 32537215

[18] Bai Z, Yao C, Zhu J, et al. Anti-tumor drug discovery based on natural product *β*-elemene: anti-tumor mechanisms and structural modification. Molecules 2021;26:1499. 33801899 10.3390/molecules26061499 PMC7998186

[19] Chen J, Wang R, Wang T, et al. Antioxidant properties of novel dimers derived from natural *β*-elemene through inhibiting H_2_O_2_-induced apoptosis. ACS Med Chem Lett 2017;8:443–8. 28435534 10.1021/acsmedchemlett.7b00035 PMC5392764

[20] Chen J, Wang T, Xu S, et al. Novel hybrids of natural *β*-elemene bearing isopropanolamine moieties: Synthesis, enhanced anticancer profile, and improved aqueous solubility. Fitoterapia 2017;120:117–25. 28576721 10.1016/j.fitote.2017.05.002

[21] Chen J, Wang T, Xu S, et al. Discovery of novel anti-tumor nitric oxide-donating *β*-elemene hybrids through inhibiting the PI3K/Akt pathway. Eur J Med Chem 2017;135:414–23. 28463784 10.1016/j.ejmech.2017.04.045

[22] Bai R, Zhu J, Bai Z, et al. Second generation *β*-elemene nitric oxide derivatives with reasonable linkers: potential hybrids against malignant brain glioma. J Enzym Inhib Med Chem 2022;37:379–85. 10.1080/14756366.2021.2016734 PMC8757613 35012394

[23] Gao Y, Zhang H, Lirussi F, et al. Dual inhibitors of histone deacetylases and other cancer-related targets: a pharmacological perspective. Biochem Pharmacol 2020;182:114224. 32956642 10.1016/j.bcp.2020.114224

[24] He X, Li Z, Zhuo X-T, et al. Novel selective histone deacetylase 6 (HDAC6) inhibitors: a patent review (2016-2019). Recent Pat anti-Cancer Drug Dis 2020;15:32–48. 10.2174/1574892815666200217125419 32065106

[25] Xie T, Ye X, Li Z, et al. 2022. *β*-Elemene halide and preparation method thereof. CN 110683932 A.

