# Supplementary material for: Novel hydroxyl carboximates derived from β-elemene: design, synthesis and anti-tumour activities evaluation
Source: J Enzyme Inhib Med Chem. 2022 Sep 6;37(1):2403–16. doi: 10.1080/14756366.2022.2117314 (PMC9467605; doi:10.1080/14756366.2022.2117314)
Supplement: Supplemental Material [file IENZ_A_2117314_SM1942.pdf]

Supporting Information

Novel hydroxyl carboximates derived from β-elemene: design, synthesis and anti-tumor activities evaluation

Yuan Gao [a, b, c, d, e], Nian-Dong Mao [a, b, c, d], Li Xu [a, b, c, d], Renren Bai [a, b, c, d], Li-Wei Wang [a, b, c, d, *], Xiang-Yang Ye [a, b, c, d, *], Tian Xie [a, b, c, d, e *]

[a]*School of Pharmacy, Hangzhou Normal University, Hangzhou, Zhejiang 311121, China*

[b]*Key Laboratory of Elemene Class Anti-Cancer Chinese Medicine of Zhejiang Province, China*

[c]*Engineering Laboratory of Development and Application of Traditional Chinese Medicine from Zhejiang Province, China*

[d]*Collaborative Innovation Center of Chinese Medicines from Zhejiang Province, China*

[e]*Institute of Chinese Materia Medical, Shanghai University of Traditional Chinese Medicine, Shanghai 200000, China*

Contents

^{1}H and ^{13}C NMR spectra of compounds **11a-f**, **11h-11j**, **15** and **17**

HPLC spectra of compounds **11a-f**, **11h-11j**, **15** and **17**

Information Classification: General

^{1}H and ^{13}C NMR spectra of compounds 11a-f, 11h-11j, 15 and **17**

6-Amino-*N*-hydroxy-*N*-(2-((1*R*,3*S*,4*S*)-4-methyl-3-(prop-1-en-2-yl)-4-vinylcyclohexyl)allyl)nicotinamide (**11a**)

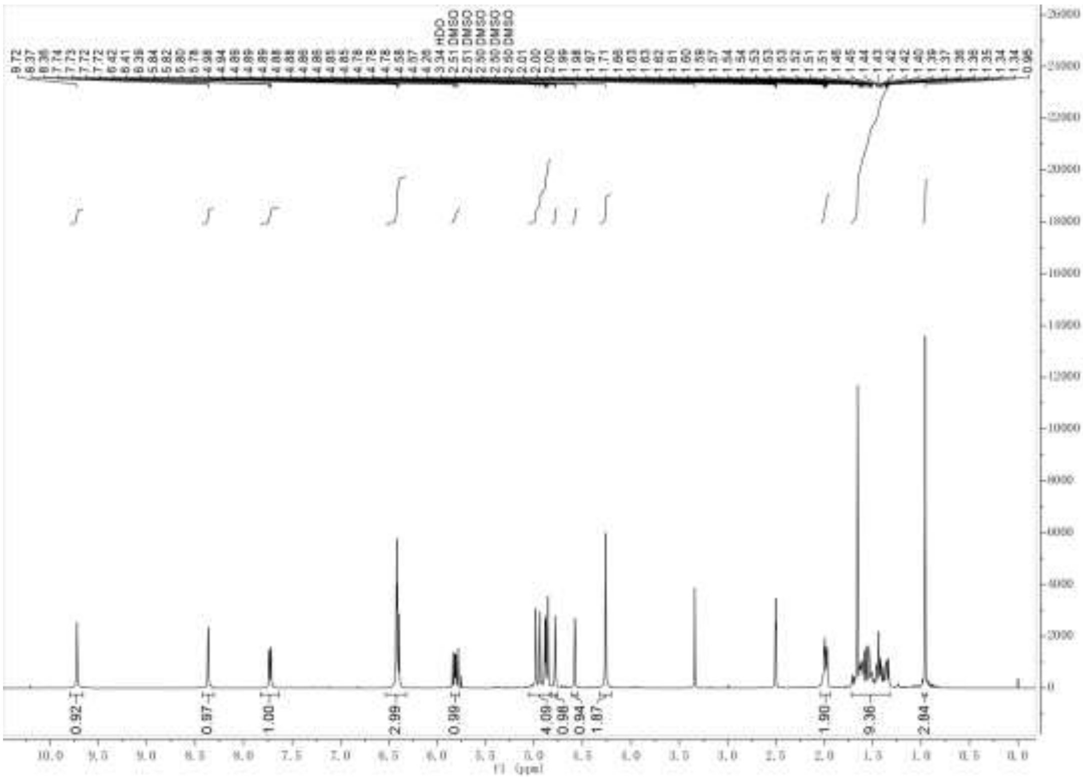

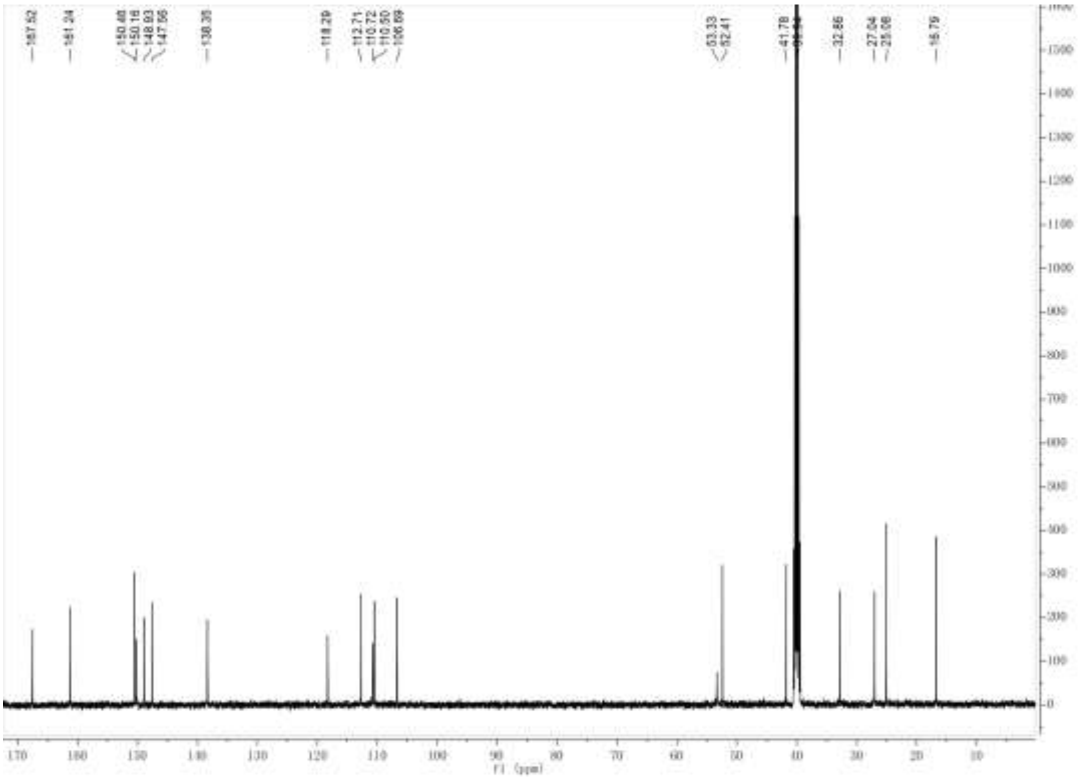

Information Classification: General

Information Classification: General

(*E*)-*N*-Hydroxy-*N*-(2-((1*R*,3*S*,4*S*)-4-methyl-3-(prop-1-en-2-yl)-4-vinylcyclohexyl)allyl)-3-(*p*-tolyl)acrylamide (**11b**)

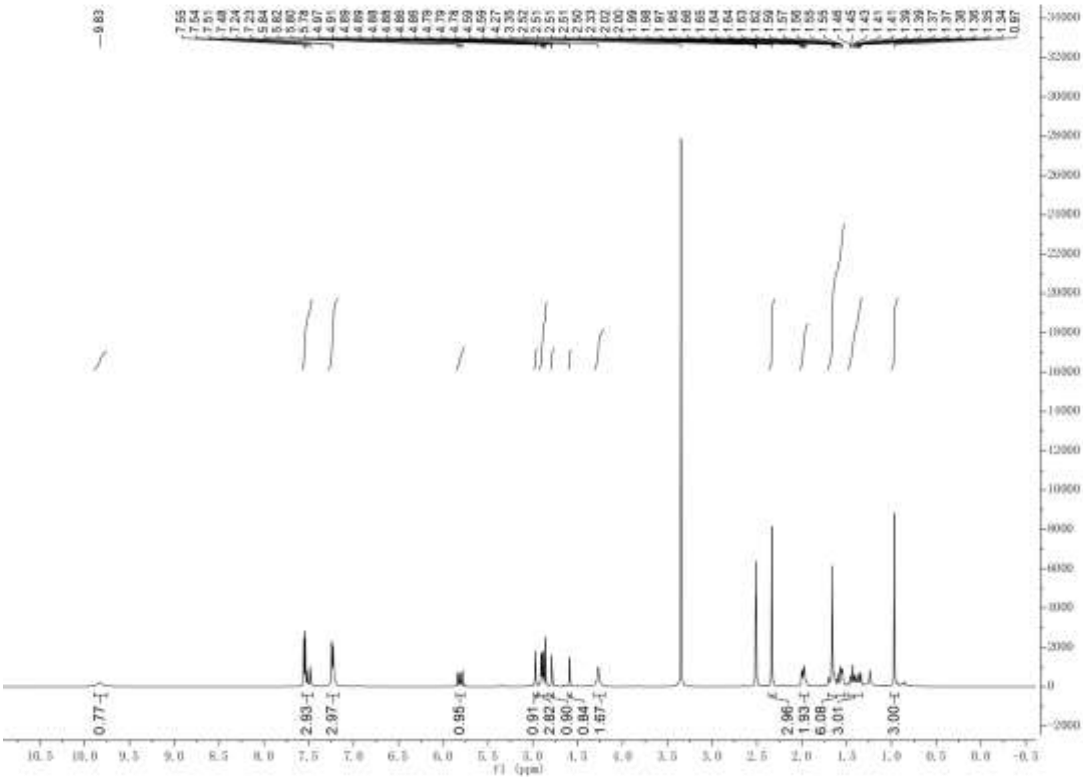

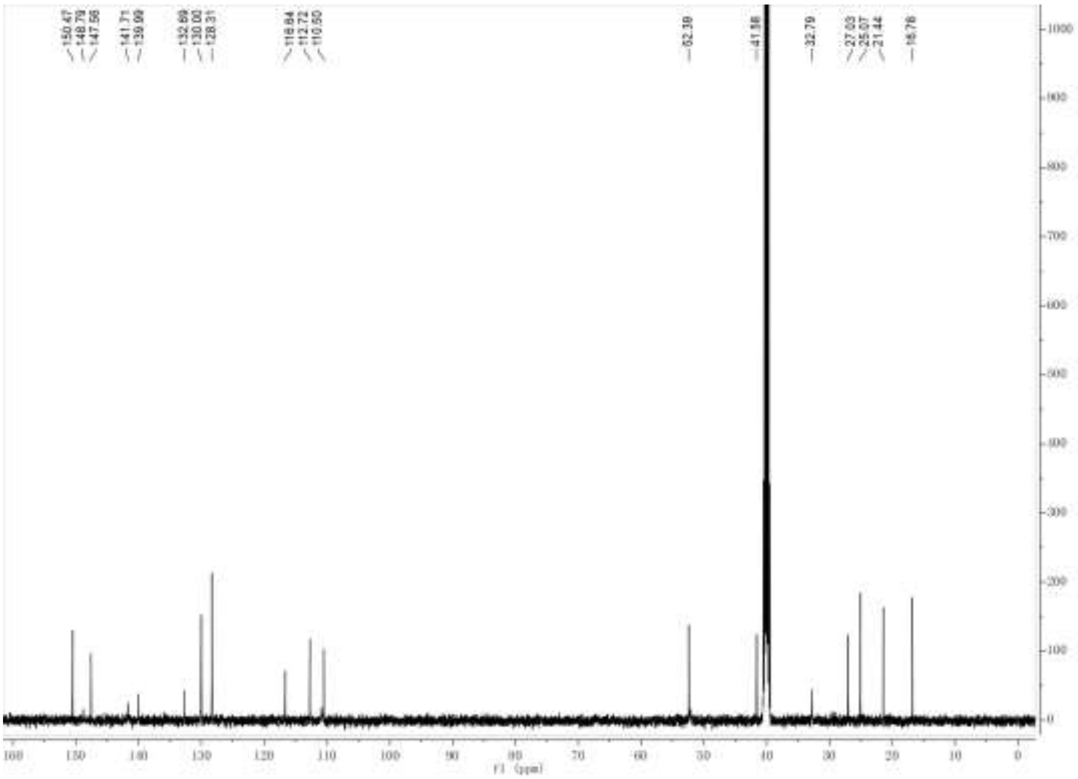

Information Classification: General

(*E*)-*N*-Hydroxy-N-(2-((1*R*,3*S*,4*S*)-4-methyl-3-(prop-1-en-2-yl)-4-vinylcyclohexyl)allyl)-3-(*m*-tolyl)acrylamide (**11c**)

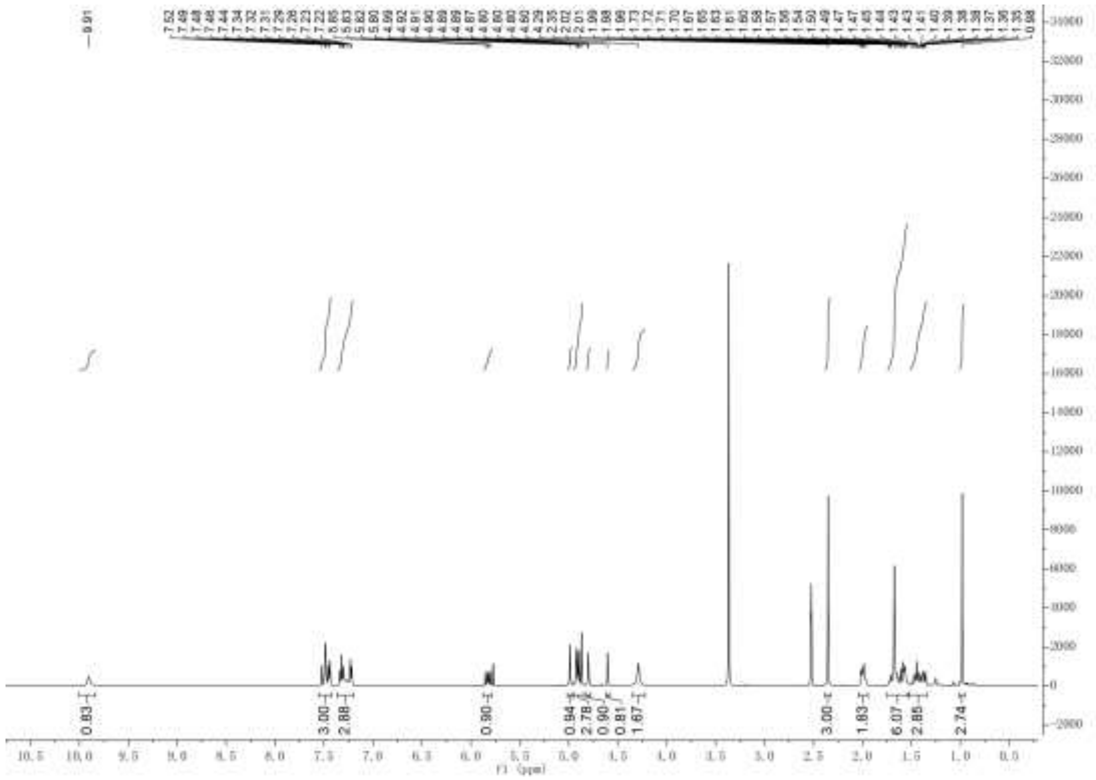

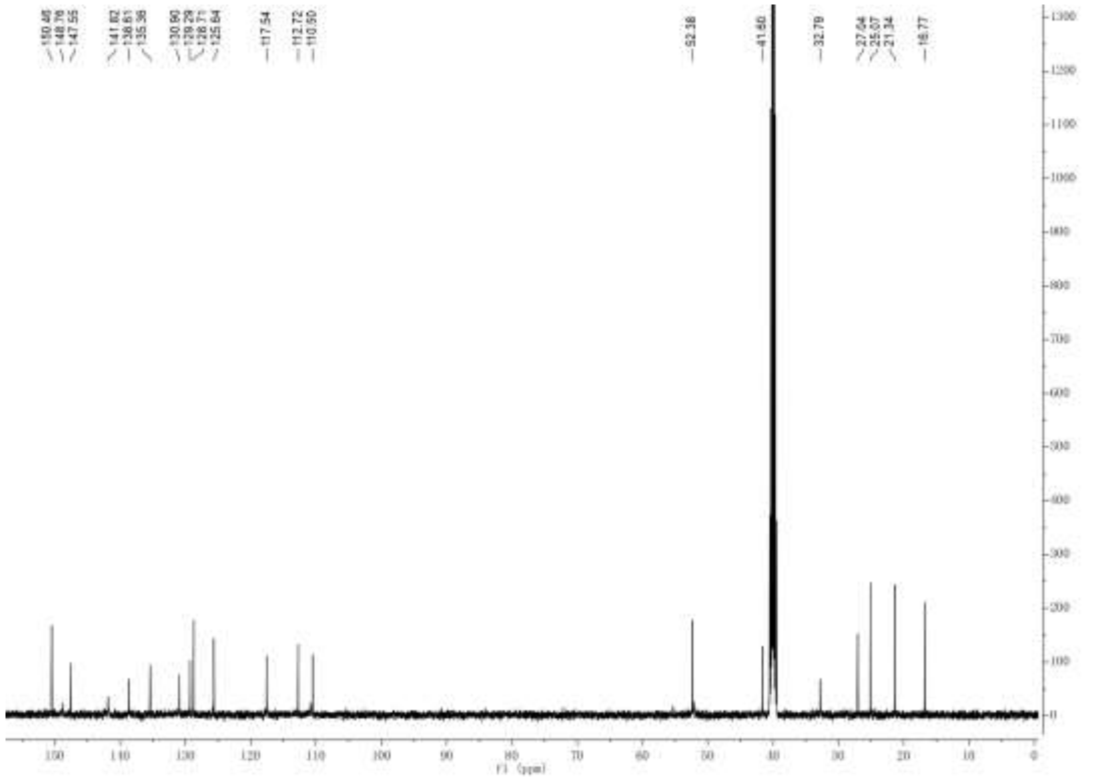

(*E*)-*N*-Hydroxy-*N*-(2-((1*R*,3*S*,4*S*)-4-methyl-3-(prop-1-en-2-yl)-4-vinylcyclohexyl)allyl)-3-(*o*-tolyl)acrylamide (**11d**)

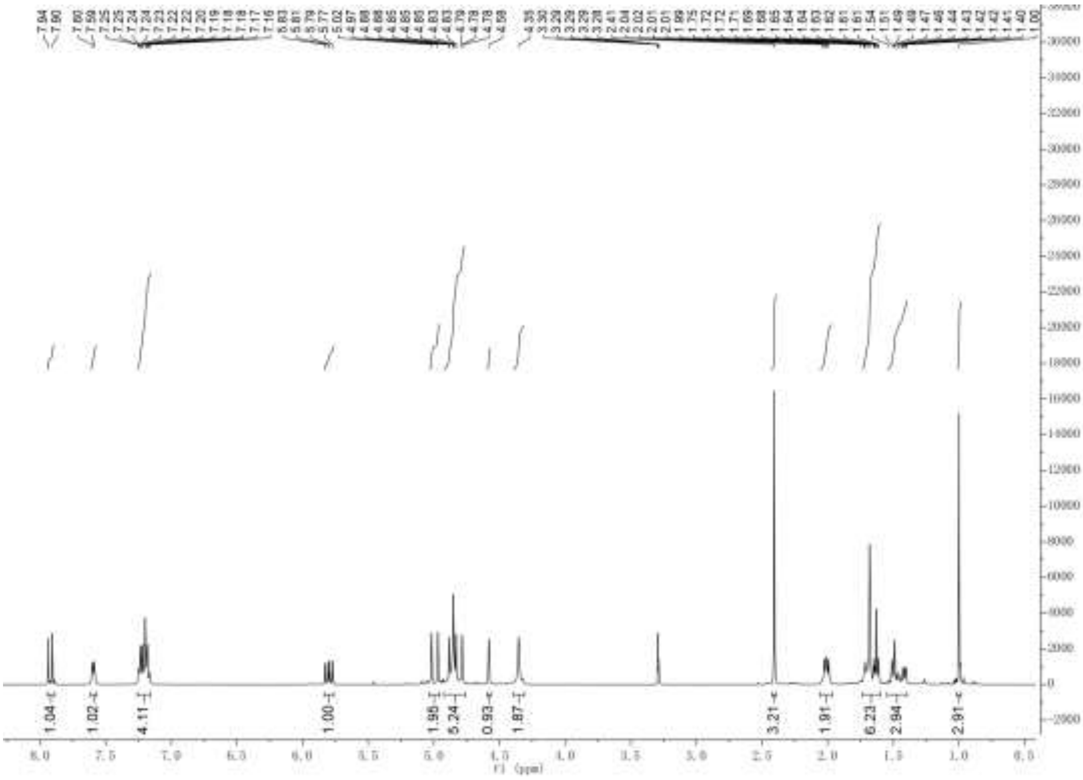

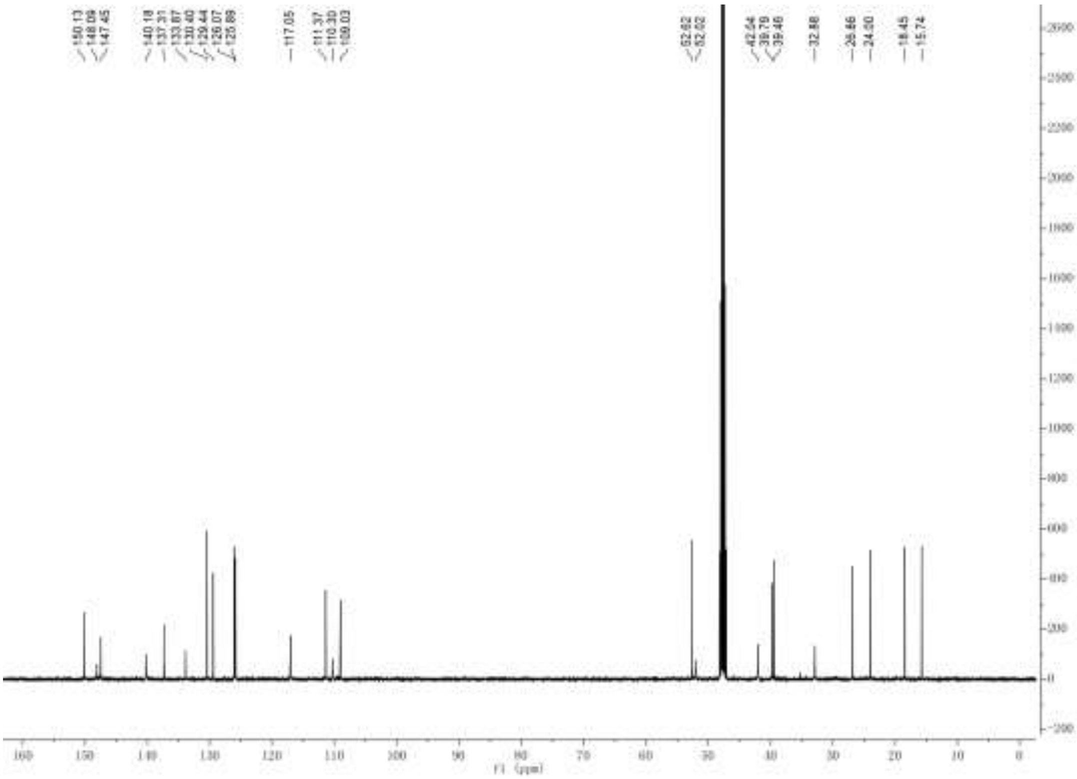

Information Classification: General

(*E*)-3-(4-Cyanophenyl)-*N*-hydroxy-*N*-(2-((1*R*,3*S*,4*S*)-4-methyl-3-(prop-1-en-2-yl)-4-vinylcyclohexyl)allyl)acrylamide (**11e**)

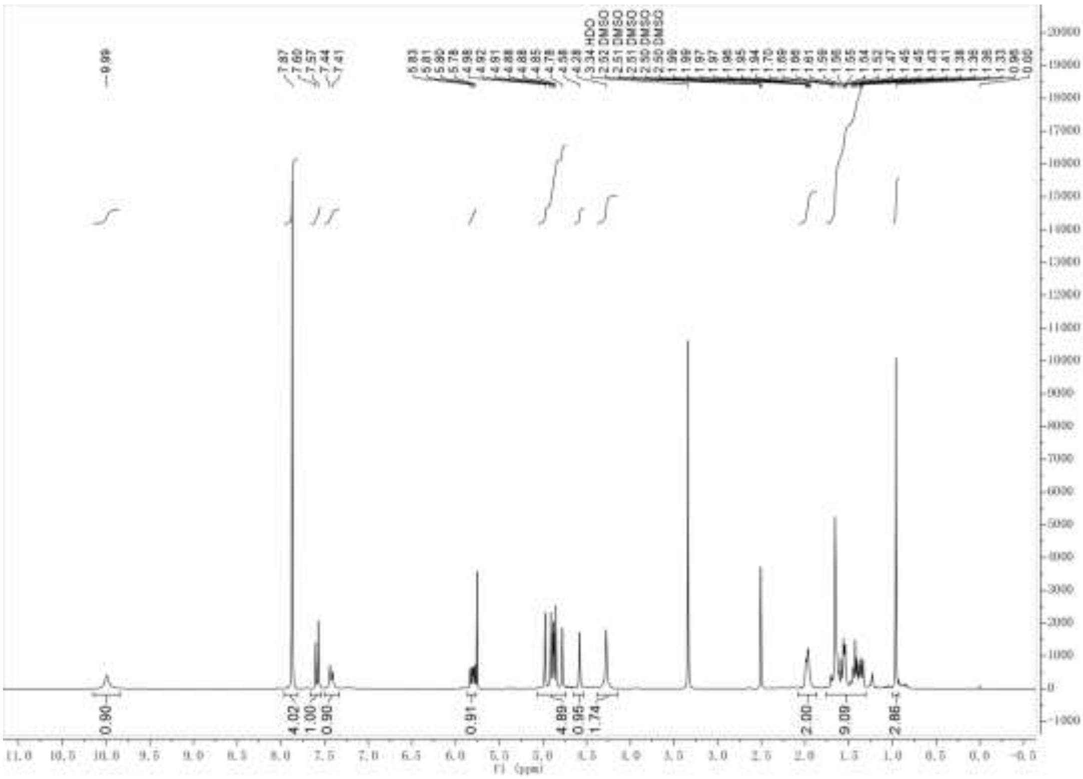

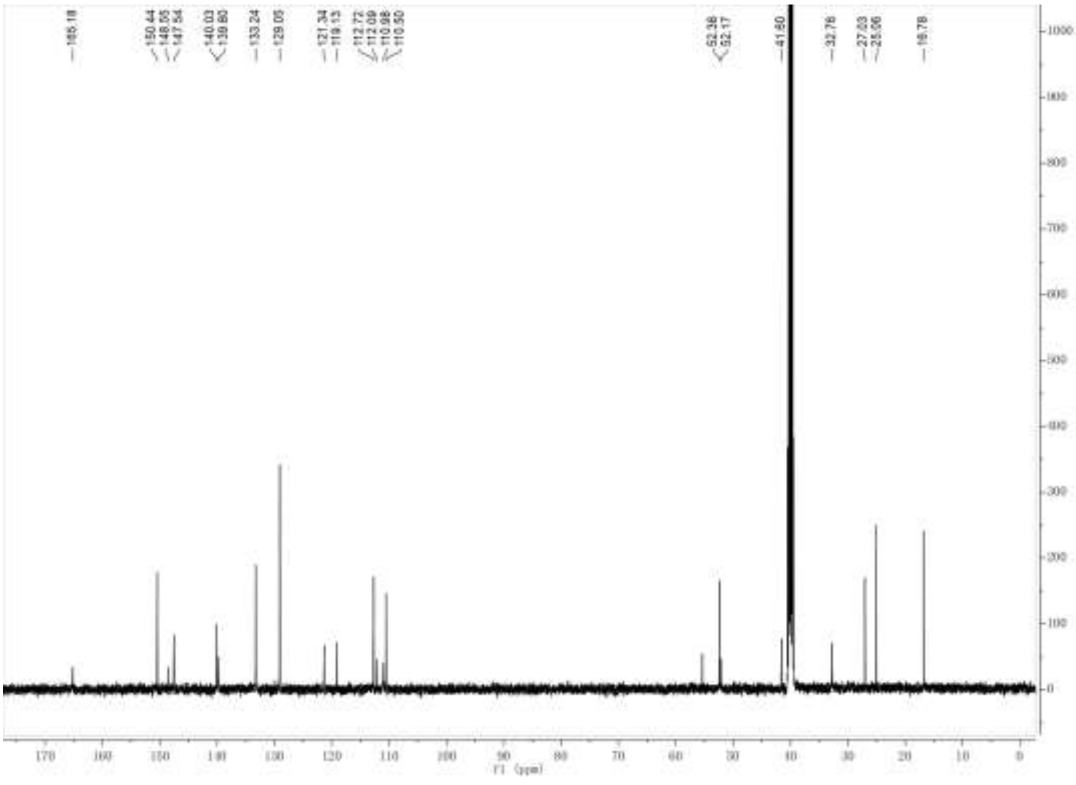

Information Classification: General

(*E*)-*N*-Hydroxy-*N*-(2-((1*R*,3*S*,4*S*)-4-methyl-3-(prop-1-en-2-yl)-4-vinylcyclohexyl)allyl)-3-(4-(trifluoromethyl)phenyl)acrylamide (**11f**)

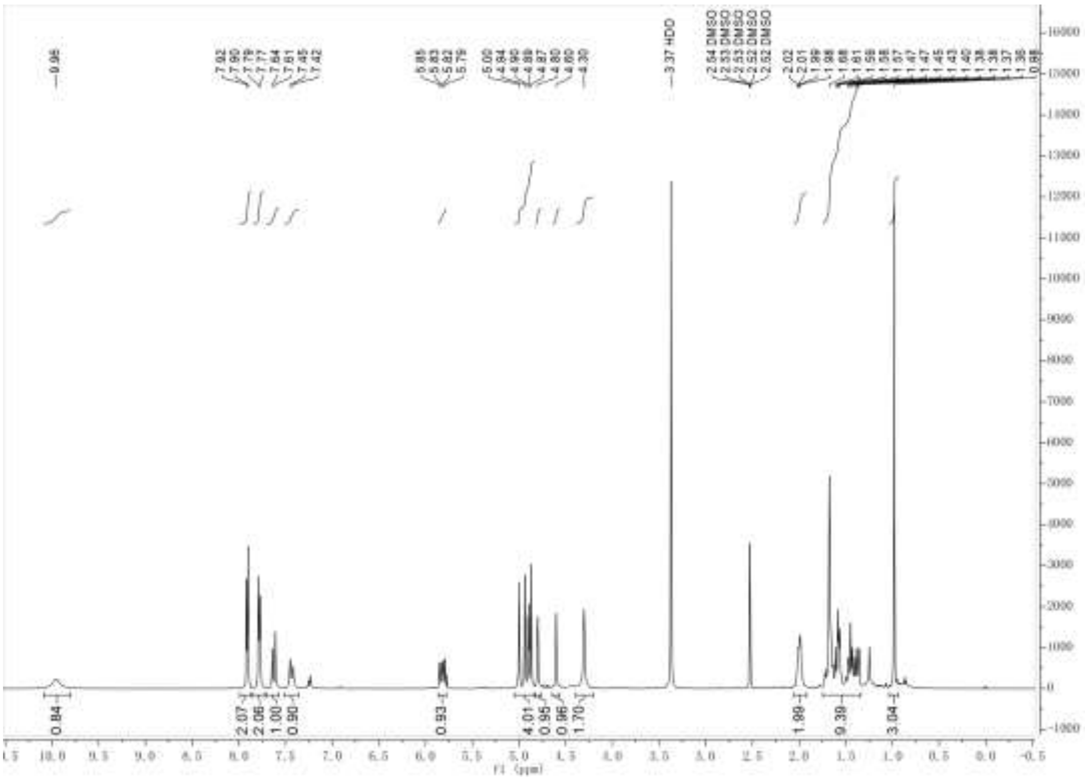

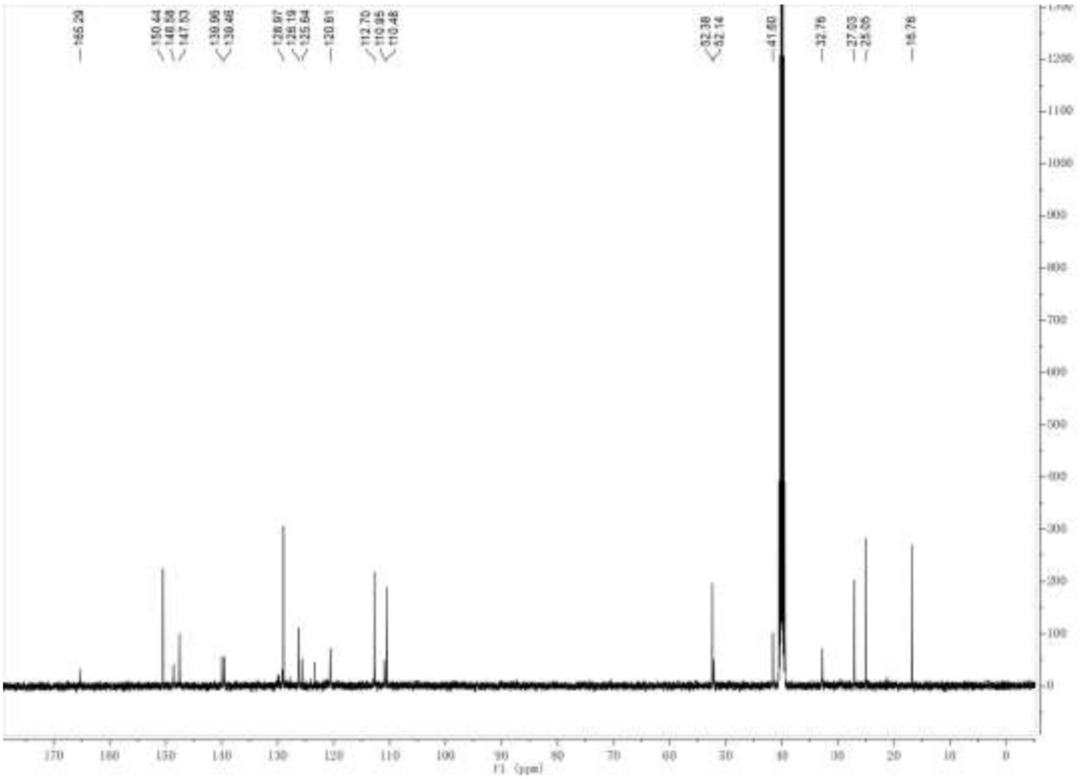

(*E*)-3-(4-Aminophenyl)-*N*-hydroxy-*N*-(2-((1*R*,3*S*,4*S*)-4-methyl-3-(prop-1-en-2-yl)-4-vinylcyclohexyl)allyl)acrylamide (**11h**)

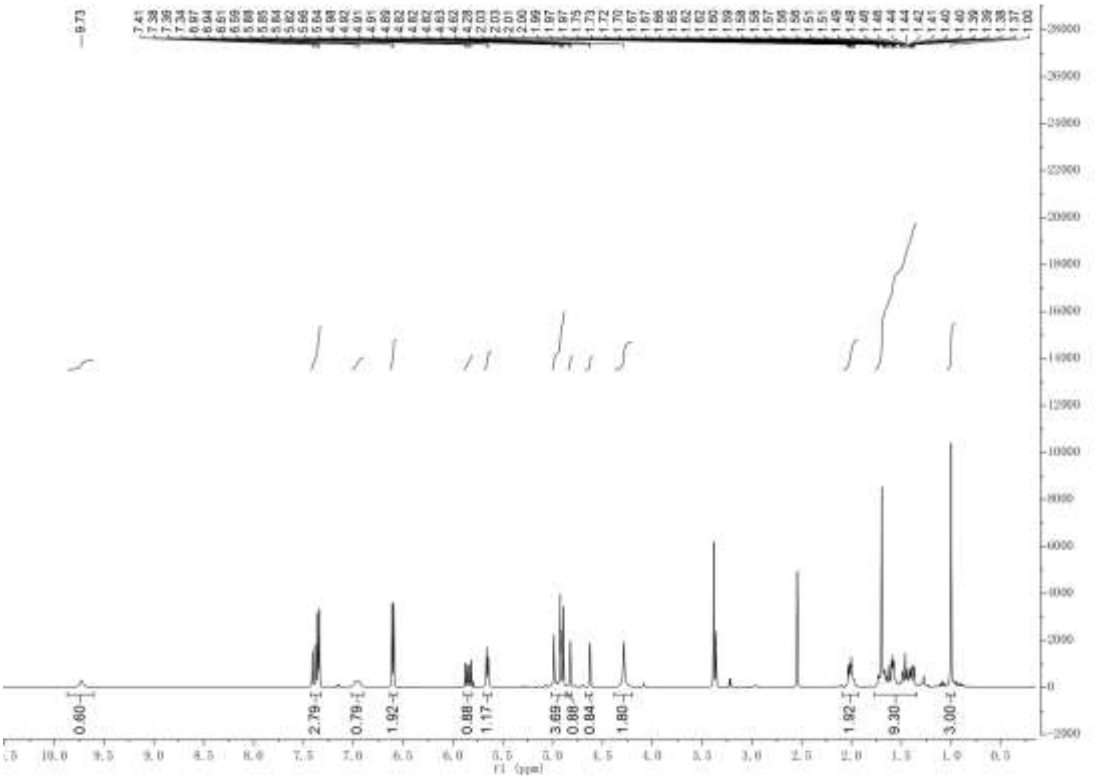

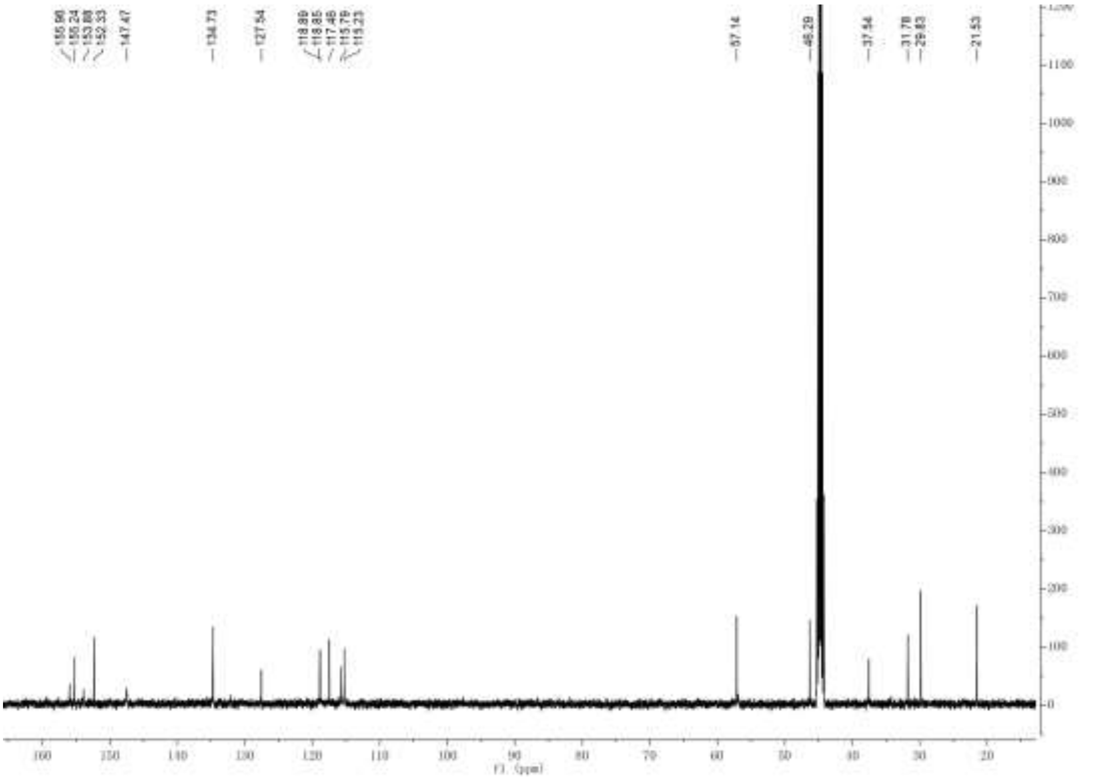

(*E*)-3-(6-Aminopyridin-3-yl)-*N*-hydroxy-*N*-(2-((1*R*,3*S*,4*S*)-4-methyl-3-(prop-1-en-2-yl)-4-vinylcyclohexyl)allyl)acrylamide (**11i**)

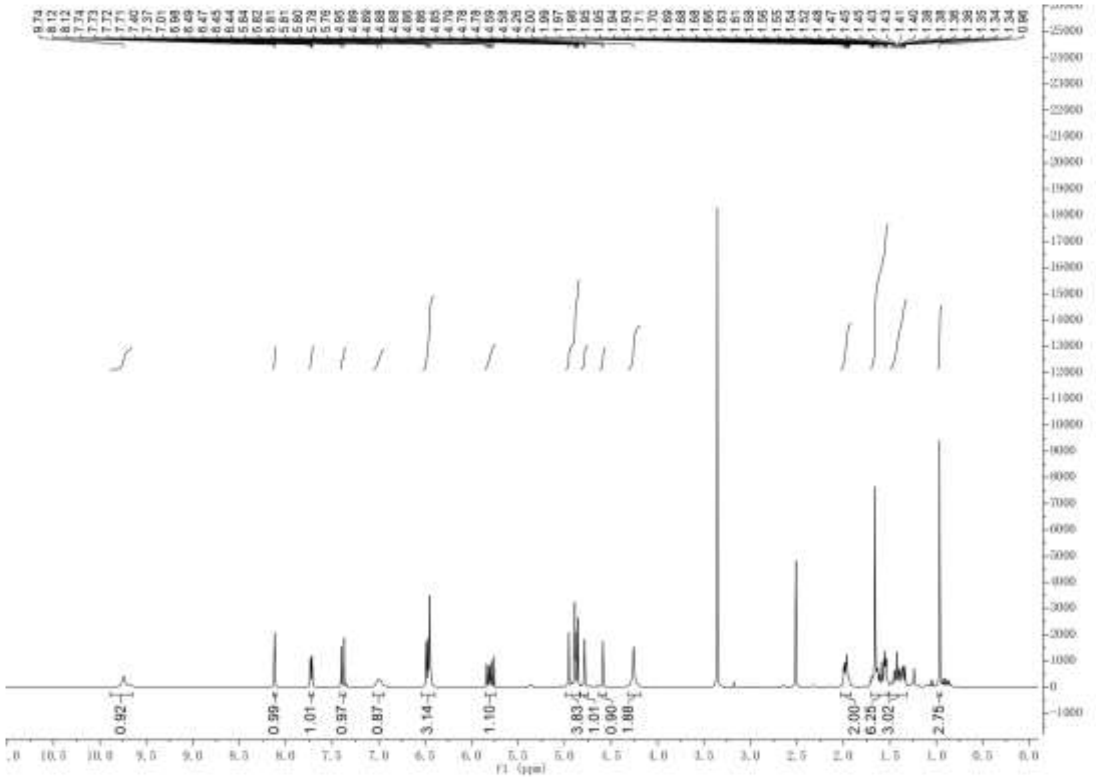

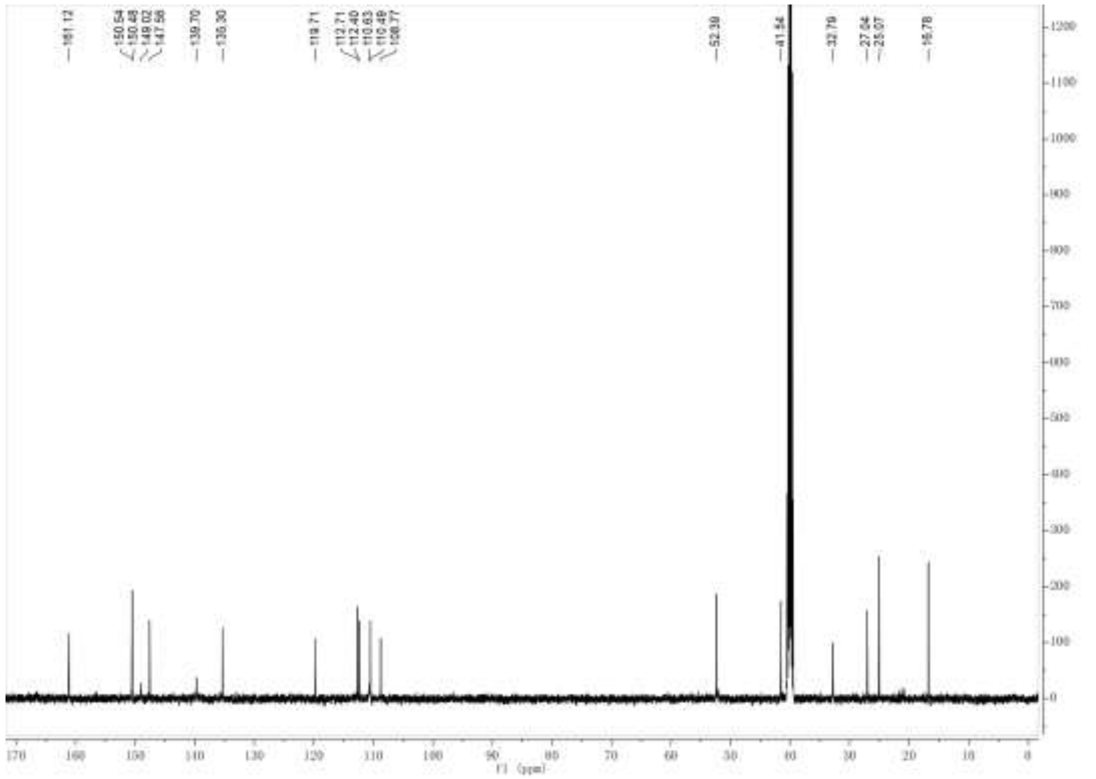

(*E*)-*N*-Hydroxy-*N*-(2-((1*R*,3*S*,4*S*)-4-methyl-3-(prop-1-en-2-yl)-4-vinylcyclohexyl)allyl)-3-(pyridin-3-yl)acrylamide (**11j**)

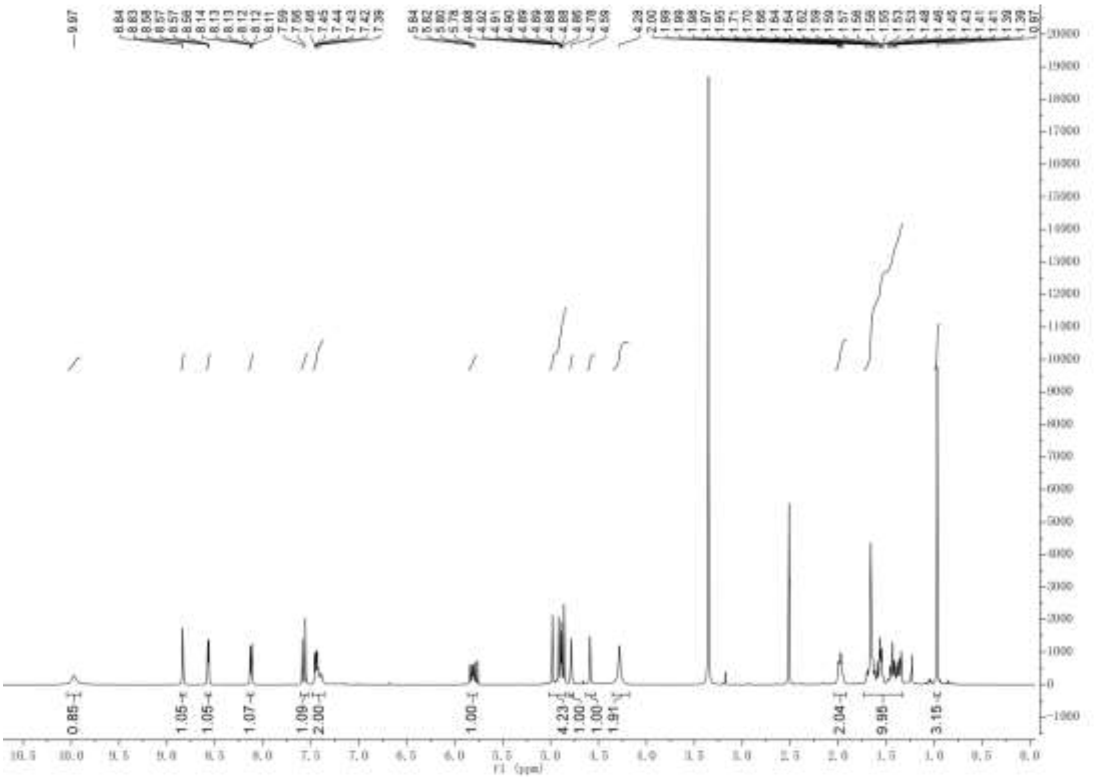

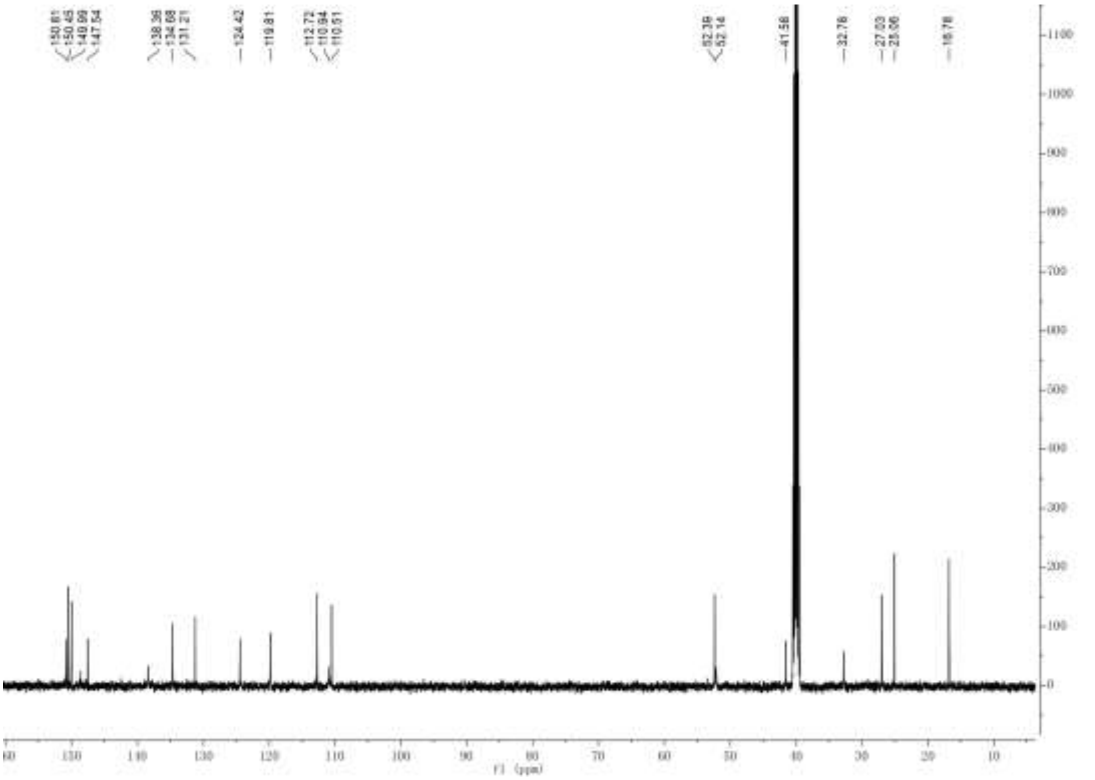

Information Classification: General

(*E*)-3-(6-Aminopyridin-3-yl)-*N*-cyclopropyl-*N*-(2-((1*R*,3*S*,4*S*)-4-methyl-3-(prop-1-en-2-yl)-4-vinylcyclohexyl)allyl)acrylamide (**15**)

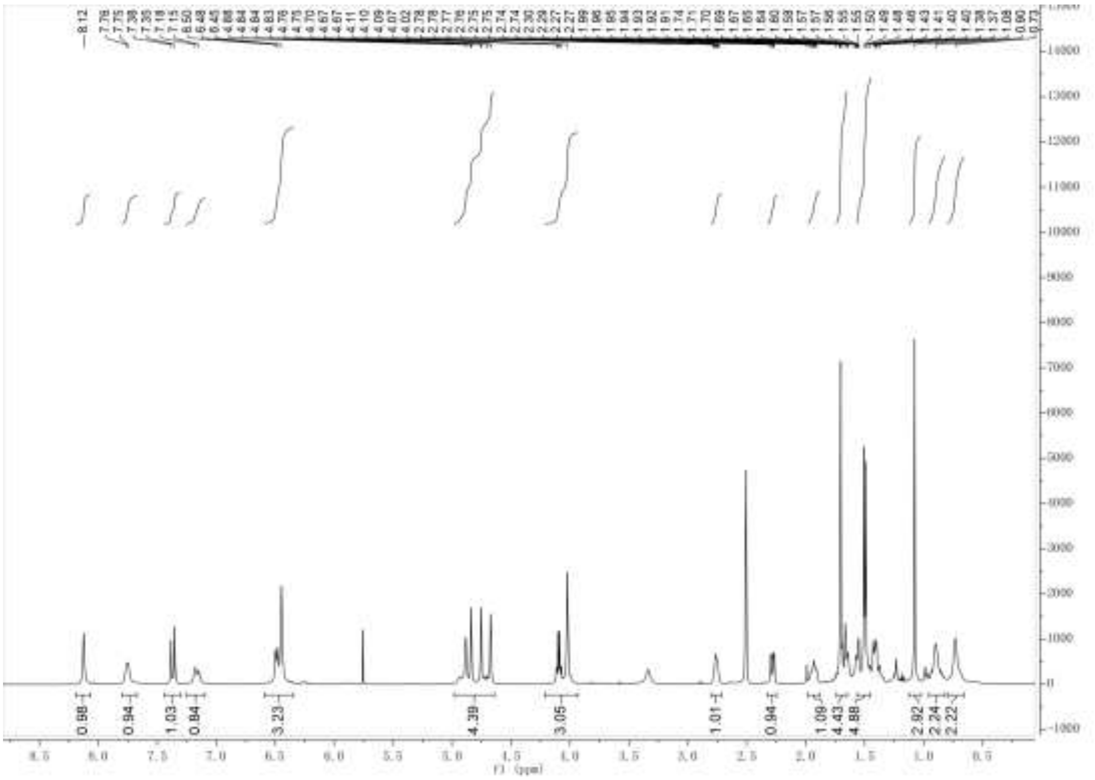

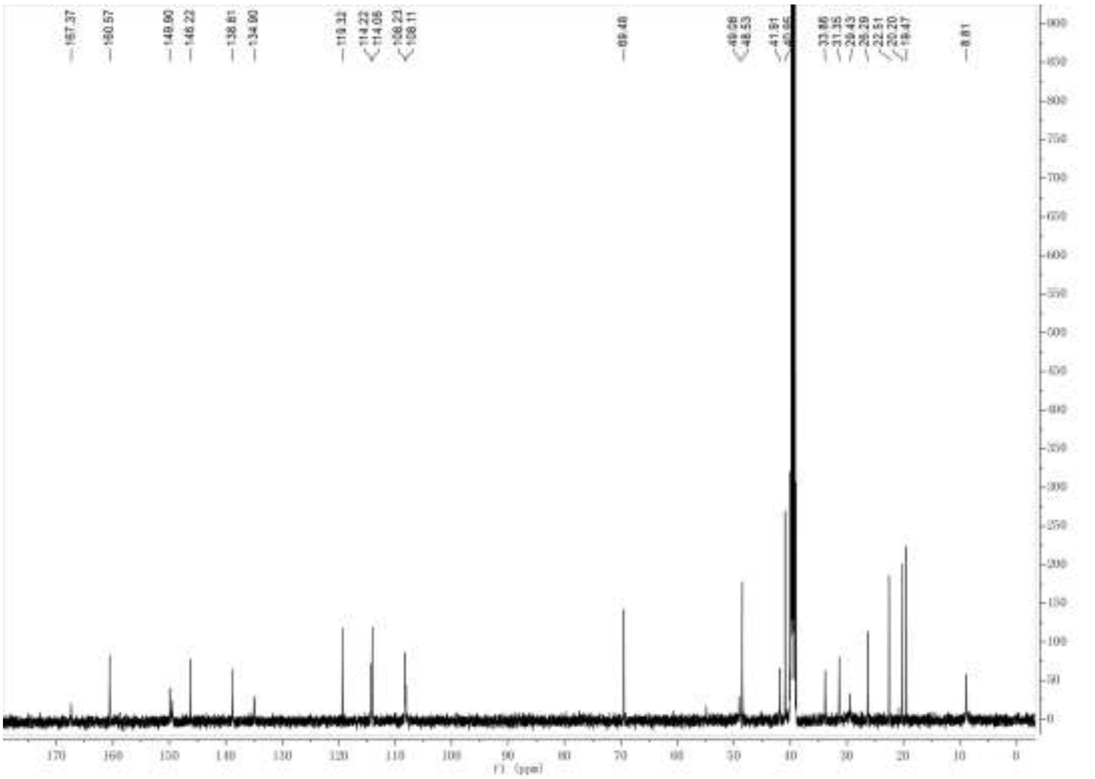

(*E*)-*N*-Hydroxy-*N*-(2-((1*R*,3*S*,4*S*)-4-methyl-3-(prop-1-en-2-yl)-4-vinylcyclohexyl)allyl)-3-(4-((2-((1*R*,3*S*,4*S*)-4-methyl-3-(prop-1-en-2-yl)-4-vinylcyclohexyl)allyl)oxy)phenyl)acrylamide (**17**)

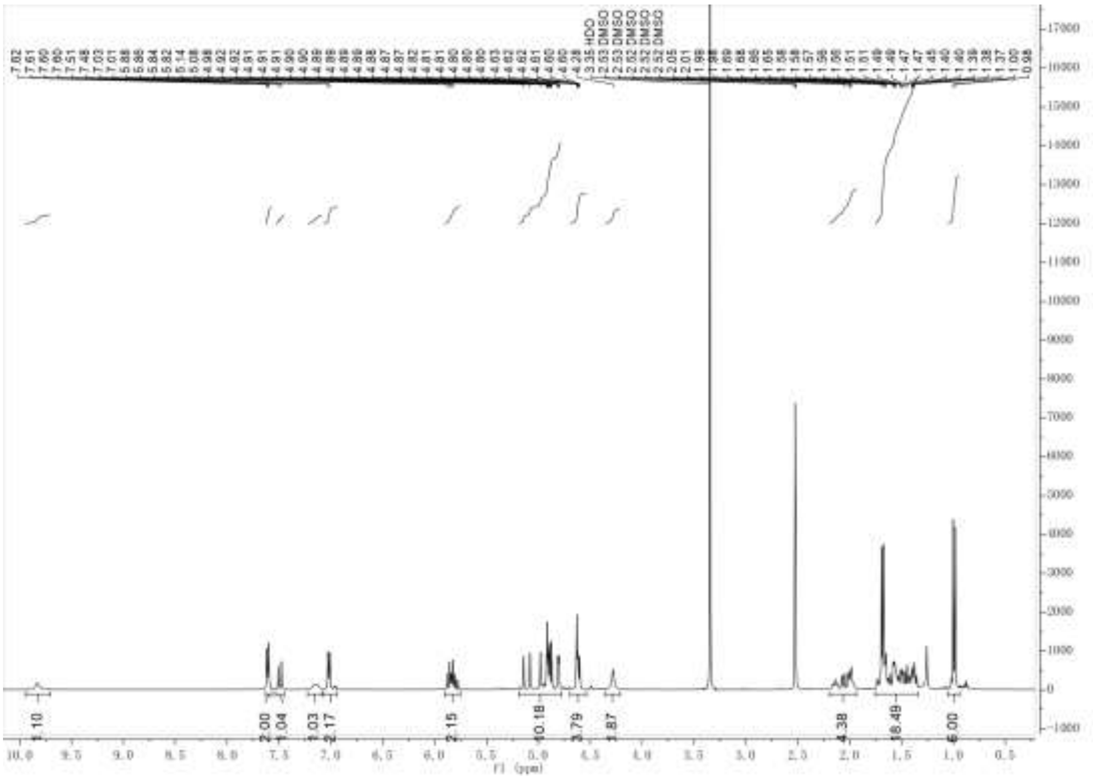

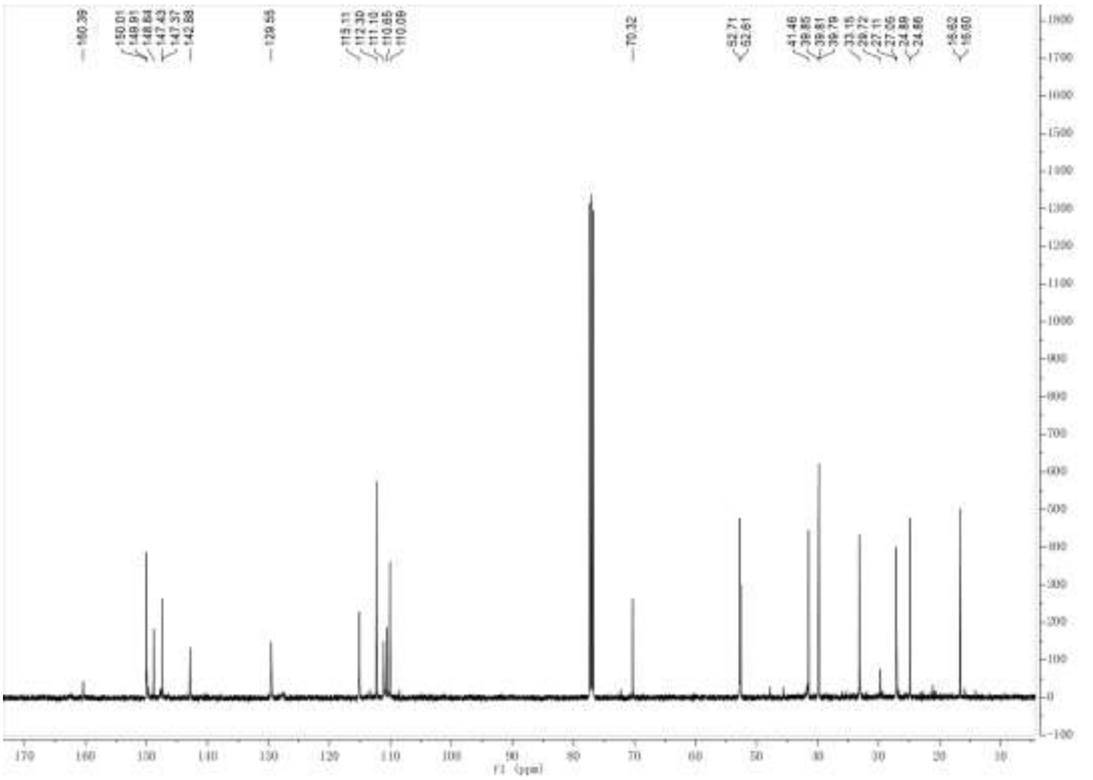

Information Classification: General

HPLC spectra of compounds 11a-f, 11h-11j, 15 and 17

HPLC spectra of compound **11a** (purity: 98.91%)

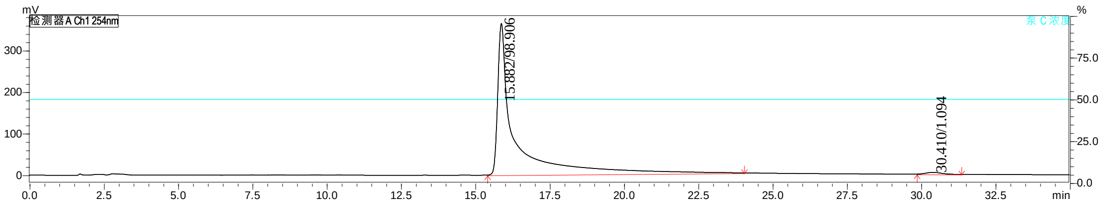

HPLC spectra of compound **11b** (purity: 95.46%)

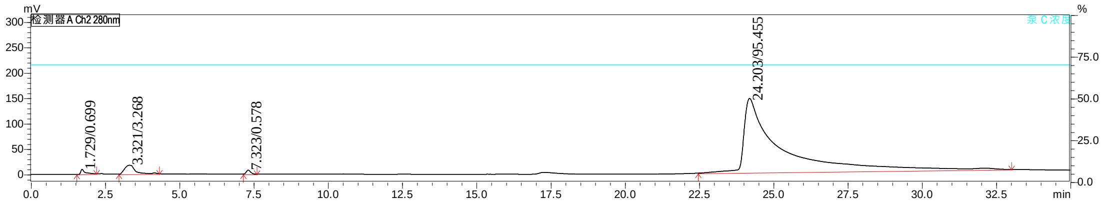

HPLC spectra of compound **11c** (purity: 98.74%)

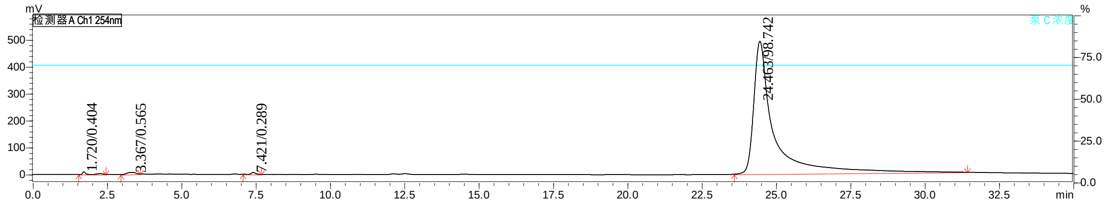

HPLC spectra of compound **11d** (purity: 99.70%)

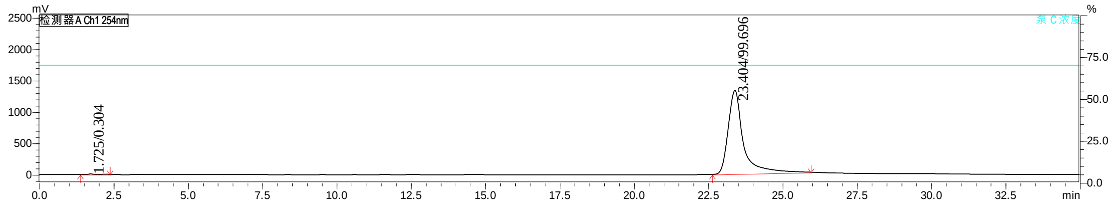

HPLC spectra of compound **11e** (purity: 91.98%)

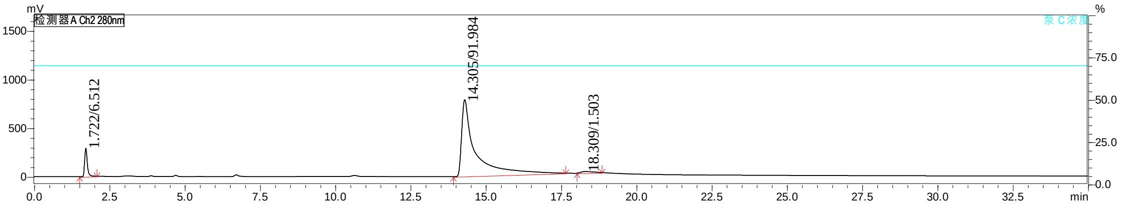

Information Classification: General

HPLC spectra of compound **11f** (purity: 92.37%)

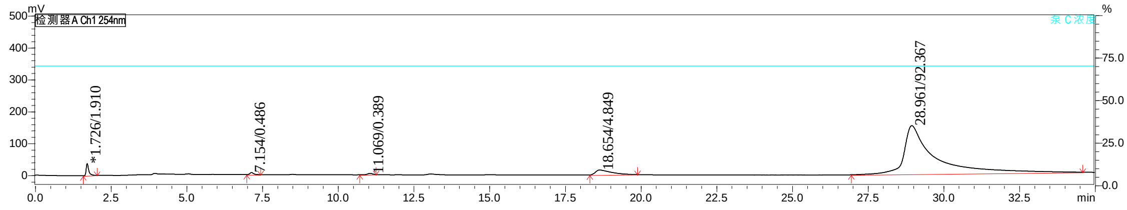

HPLC spectra of compound **11h** (purity: 98.86%)

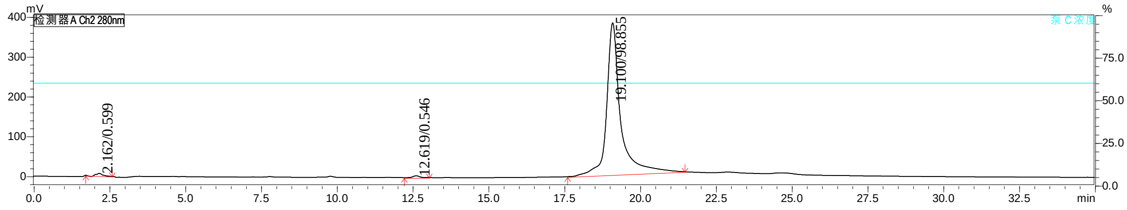

HPLC spectra of compound **11i** (purity: 99.13%)

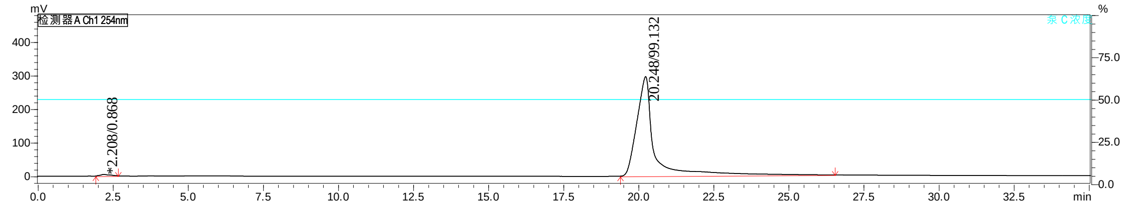

HPLC spectra of compound **11j** (purity: 97.74%)

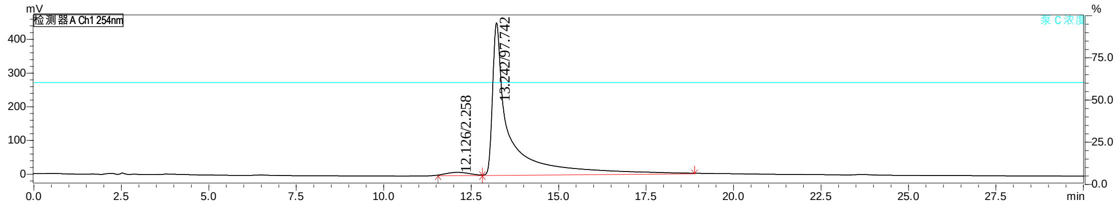

HPLC spectra of compound **15** (purity: 94.27%)

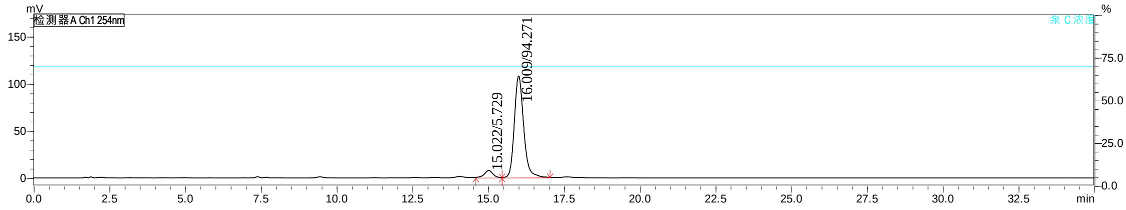

HPLC spectra of compound **17** (purity: 97.60%)

Information Classification: General

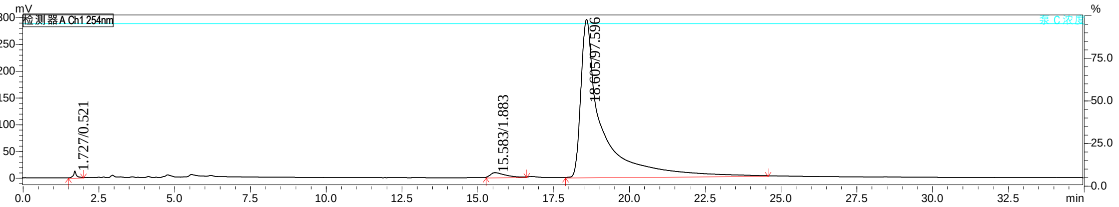

Information Classification: General
